# High-energy-level metabolism and transport occur at the transition from closed to open flowers

**DOI:** 10.1093/plphys/kiac253

**Published:** 2022-05-28

**Authors:** Monica Borghi, Leonardo Perez de Souza, Takayuki Tohge, Jianing Mi, Giovanni Melandri, Sebastian Proost, Marina C M Martins, Salim Al-Babili, Harro J Bouwmeester, Alisdair R Fernie

**Affiliations:** Department of Biology, Utah State University, Logan, Utah 84321-5305, USA; Max Planck Institute of Molecular Plant Physiology, 14476 Potsdam, Germany; Laboratory of Plant Physiology, Wageningen University and Research, Wageningen 6708 PB, The Netherlands; Max Planck Institute of Molecular Plant Physiology, 14476 Potsdam, Germany; Max Planck Institute of Molecular Plant Physiology, 14476 Potsdam, Germany; Nara Institute of Science and Technology, Nara 630-0192, Japan; The Bioactives Lab, Biological and Environmental Sciences and Engineering Division, King Abdullah University of Science and Technology, Thuwal 23955-6900, Saudi Arabia; Laboratory of Plant Physiology, Wageningen University and Research, Wageningen 6708 PB, The Netherlands; INRAE, University of Bordeaux, UMR BFP, Villenave d’Ornon 33140, France; Max Planck Institute of Molecular Plant Physiology, 14476 Potsdam, Germany; Laboratory of Molecular Bacteriology, Department of Microbiology and Immunology, Rega Institute, KU Leuven, Leuven, Belgium; Center for Microbiology, VIB, Leuven 3000, Belgium; Max Planck Institute of Molecular Plant Physiology, 14476 Potsdam, Germany; In Press—Consultoria e Comunicação Científica, São Paulo 05089-030, Brazil; The Bioactives Lab, Biological and Environmental Sciences and Engineering Division, King Abdullah University of Science and Technology, Thuwal 23955-6900, Saudi Arabia; Laboratory of Plant Physiology, Wageningen University and Research, Wageningen 6708 PB, The Netherlands; Swammerdam Institute for Life Sciences, University of Amsterdam, 1098 XH Amsterdam, The Netherlands; Max Planck Institute of Molecular Plant Physiology, 14476 Potsdam, Germany

## Abstract

During the maturation phase of flower development, the onset of anthesis visibly marks the transition from buds to open flowers, during which petals stretch out, nectar secretion commences, and pollination occurs. Analysis of the metabolic changes occurring during this developmental transition has primarily focused on specific classes of metabolites, such as pigments and scent emission, and far less on the whole network of primary and secondary metabolites. To investigate the metabolic changes occurring at anthesis, we performed multi-platform metabolomics alongside RNA sequencing in individual florets harvested from the main inflorescence of Arabidopsis (*Arabidopsis thaliana*) ecotype Col-0. To trace metabolic fluxes at the level of the whole inflorescence and individual florets, we further integrated these studies with radiolabeled experiments. These extensive analyses revealed high-energy-level metabolism and transport of carbohydrates and amino acids, supporting intense metabolic rearrangements occurring at the time of this floral transition. These comprehensive data are discussed in the context of our current understanding of the metabolic shifts underlying flower opening. We envision that this analysis will facilitate the introgression of floral metabolic traits promoting pollination in crop species for which a comprehensive knowledge of flower metabolism is still limited.

## Teaser

Anthesis is characterized by intense changes in metabolism accompanied by temporal regulation of central metabolic pathways.

## Introduction

At the end of the maturation phase, the onset of anthesis marks a time of remarkable visual transition in the life of flowers. In this stage, petals acquire their final shape and size, display their color, and start emitting scent, and in plants provided with nectaries, nectar secretion also commences. This morphological transition underlies a dynamic reorganization of the metabolism of flowers, obtained, among others, with increased synthesis and accumulation of pigments and emission of volatile organic compounds (VOCs). Depolymerization of complex carbohydrates also occurs at anthesis and it is followed by the export of mono and disaccharides to the apoplast which are later found in nectar (Roy et al., 2017). Simple sugars also accumulate in the vacuole as they contribute to increasing the osmotic potential of the cells of flowers and propel floral bud opening and growth by cell expansion and elongation (van Doorn and Van Meeteren, 2003). Pollination and fertilization, which also occur at anthesis, or soon after anthesis, are similarly driven by changes in the metabolism of flowers and the metabolic signaling, which despite remaining unseen, support the interaction between pollen and stigma and the growth of the pollen tube along the transmitting tissue (Rounds et al., 2011).

Flowers have extensively been utilized for interrogating processes of biosynthesis and accumulation of secondary metabolites and their physiological function. For example, flowers of petunia (*Petunia hybrida*) have served as a model to investigate the regulation of VOC synthesis and emission. In these flowers, where scent emission starts at anthesis and continues with nocturnal oscillations until withering, it was shown that genes codifying for enzymes of VOC synthesis are transcriptionally upregulated as the corolla opens and downregulated after pollination (Fenske et al., 2015; Lynch et al., 2020). Biochemical analyses which extended beyond VOCs were performed in the corolla of *Nicotiana attenuata* flowers and revealed that the rates of carbohydrate metabolism also increase at anthesis (Stitz et al., 2014). Similar analyses performed in petals of snapdragon (*Antirrhinum majus*) flowers revealed that the biochemical precursors of scent compounds become insufficient in postanthesis (Muhlemann et al., 2014). Ethylene (Colquhoun et al., 2010; Liu et al., 2017), gibberellic (GA), and jasmonic (JA) acids (Hong et al., 2012; Stitz et al., 2014) collectively act as signals to promote or cease VOC emission. However, it is currently unknown whether a progressive quenching of primary metabolism or redirection of the metabolic flux away from pathways of VOC synthesis is inducive of reduced scent emission observed in postanthesis. Flowers of Petunia also served as a model to study the molecular mechanisms of pigment synthesis and accumulation. Comparative measurements of transcript abundance in wild-type and silenced lines revealed that pigmentation and patterning in the corolla of many flowers are primarily driven by transcription (Tornielli et al., 2009; Stracke et al., 2010; Shan et al., 2020), and nowadays, numerous transcription factors (TFs) regulating the activity of enzymes along the pathway of flavonoid biosynthesis have already been identified (Tornielli et al., 2009). The observation that in many species the color of flowers fades soon after pollination (Zipor et al., 2015) also suggests that the carbon resources initially allocated to pigment synthesis are potentially recycled and reallocated to support embryo development (Pélabon et al. 2015). Still, whether the flux from primary metabolism toward the synthesis of pigments is quenched or redirected toward different pathways is currently poorly investigated.

While extensive studies have been conducted on the biosynthesis and emission of scent and accumulation of pigments, metabolites of the central metabolic pathways received far less attention, except for carbohydrate transporters, of which localization and physiological function in flowers is fairly known (Borghi and Fernie, 2017). Nonetheless, the studies conducted so far support the hypothesis that during the temporal progression of their development, flowers may integrate and capture signals from the whole plant to coordinatively regulate primary and secondary metabolism to successfully support pollination and reproduction. Indeed, while flowers of different species may differ in the spatial arrangement and size of their organs, pigmentation, and fragrance, the molecular and metabolic changes underlying morphogenesis and maturation are well-coordinated in flowers of all species (Shan et al., 2019).

Therefore, the high level of connectivity between primary and secondary metabolism which is suggested by current knowledge, advocates for combining metabolomics and transcriptomics approaches to capture and disentangle the metabolic complexity characterizing this developmental transition. When applied to studies of medicinal plants, surveys of the floral metabolomes have been informative in identifying the central metabolic pathways which may enhance the biosynthesis of active compounds (Jia et al., 2016; Yang et al., 2019). Similar studies performed in rice (*Oryza sativa*) across anthesis revealed the centrality of floral carbohydrate metabolism in the conferral of tolerance to drought and heat stresses (Li et al., 2015). Despite our general understanding of flower metabolism having advanced tremendously in recent years, pressure for more knowledge is demanded from disciplines complementary to plant physiology (Borghi and Fernie, 2020). Indeed, pollination biologists have a keen interest in understanding the interdependence between flower primary and secondary metabolism, given that secondary metabolites lure pollinators to flowers while primary metabolites serve as food for the animals and their brood (Borghi et al., 2017). Answers to questions about the cost of metabolite production and the source to sink redistribution of metabolites in flowers in preanthesis and postanthesis are also demanded from crop scientists who seek to gain knowledge on how flower metabolism changes in response to environmental perturbations.

To answer these questions, we took a multi-omics approach to characterize the molecular and metabolic rearrangements occurring at anthesis, from when flowers prepare to open until they wither. We performed multi-platform metabolomics, alongside RNA sequencing at eight consecutive stages of flower development spanning from Stages S9 to S16 (Smyth et al., 1990). We covered a developmental transition during which petals and male and female gametophytes acquire their final shape and function until when, after pollination had occurred, the process of flower senescence begins. To assess the source-to-sink distribution of metabolites transported into flowers from the phloem, we performed a detailed analysis of the metabolic fluxes at the level of the whole inflorescence and individual florets. Extensive analyses of the obtained results revealed high-energy-level metabolism and transport of carbohydrates and amino acids supporting intense metabolic rearrangements occurring during this floral transition. These comprehensive data are discussed in the context of our current understanding of the metabolic shifts underlying flower opening.

## Results

### Experimental design

**Figure 1:**
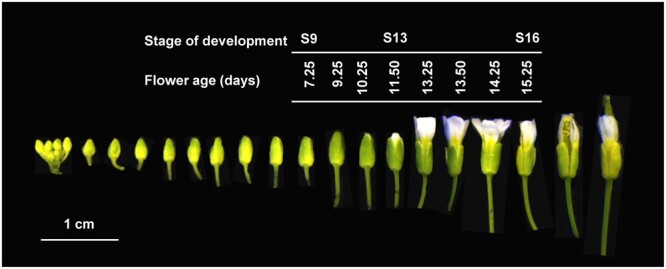
*Arabidopsis thaliana* florets arranged in a developmental progression from young (left) to old (right). The white bar marks the florets utilized in this experiment, their age (days), and stage of development accordingly to the standard nomenclature. Anthesis occurs at Stage S13. The florets were imaged at the same time and the background was removed. The bar at the bottom represents 1 cm.

To investigate the metabolic changes occurring at anthesis, we performed high-throughput metabolomics and transcriptomics in individual florets harvested from the main inflorescence of Arabidopsis (*Arabidopsis thaliana*) ecotype Col-0. Each floret was in one of the eight consecutive stages of development ranging from Stage S9 to Stage S16 (Smyth et al., 1990) to cover a temporal interval of 8 days (4 days in preanthesis and 4 days in postanthesis; Figure 1). Pools of ∼40 individual florets per stage of development were analyzed for the content of primary metabolites (sugars, amino acids, and organic acids), secondary metabolites (flavonoids, glucosinolates, polyamines, and apocarotenoids), hormones, VOCs, and transcripts, thus to gain a broad and extensive overview of the metabolic rearrangements occurring during this developmental transition.

### High steady-state level of primary metabolites and more subtle variations in the content of secondary metabolites were detected across anthesis

#### Primary metabolites

**Figure 2:**
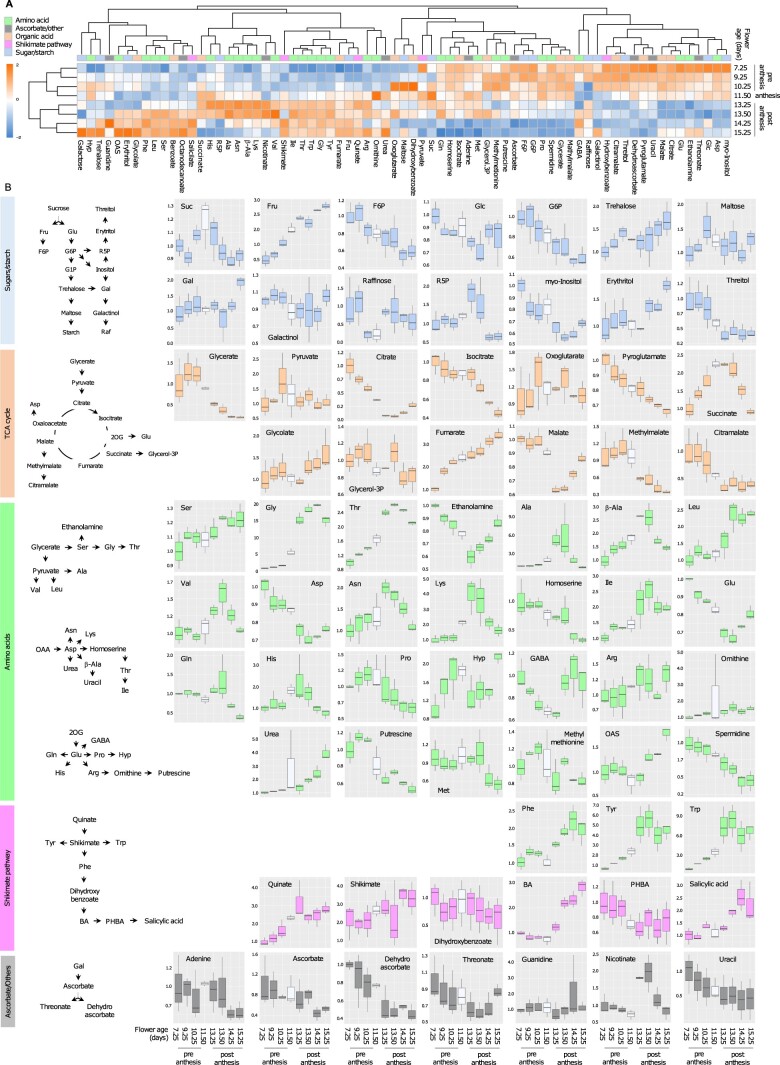
Profile of primary metabolites in *A. thaliana* florets. A, Heatmap of primary metabolites in eight stages of flower development. Florets in the stage of anthesis are 11.50 days old. Each square in the heatmap represents the log_2_ average of metabolite content from three biological replicates each composed of ∼40 florets normalized by FW and internal standard. The colored bar atop the heatmap indicates the following metabolite–pathway associations: light blue, sugar and starch metabolism; tan, TCA cycle; green, amino acids; lilac, shikimate pathway; gray, ascorbate and other compounds. B, Schematic representation of metabolic pathways (left) and boxplots (right) representing the content of primary metabolites measured in Arabidopsis florets in each of the eight stages of development relative to the youngest stage (7.29 days old buds). The boxplots show the median (central bar), the interquartile range (box), and minimum and maximum values (vertical bars). The color of the boxplots represents metabolite-pathway associations as previously described. In each square, the stage of anthesis is represented in white. 2OG, 2-Oxoglutarate; F6P, fructose-6P; G6P, glucose-6P; OAA, Oxaloacetate; OAS, *O*-acetylserine; PHBA, *p*-hydroxybenzoic acid; R5P, ribulose-5P.

After measuring the content of primary metabolites in each stage of flower development with a well-established gas-chromatography mass spectrometry (GC–MS) method (Supplemental Data Set S1), we utilized a multivariate analysis approach to assess whether metabolites display changes in abundance as flowers progress through anthesis. When we visualized the average content of primary metabolites on a heatmap, two clusters of flower samples were identified (Figure 2A): a first cluster that included floral buds and flowers at the beginning of flower opening, and a second cluster with only open flowers. Within each of these clusters, subgroups of samples with similar developmental progression were identified. Indeed, very young buds (age 7.25–9.25 days) separated from buds approaching anthesis (age 10.25–11.50 days), and young mature flowers (age 13.25–13.50 days) clustered separately from flowers entering senescence (age 14.25–15.25 days). With respect to metabolite abundance, two clusters were also observed. A first cluster which included metabolites present in high-abundance in preanthesis, of which the content decreased in postanthesis, and a second cluster was characterized by low-abundant metabolites in preanthesis, of which the content increased as flowers opened. Thus, the heatmap of primary metabolites appears partitioned in four main quarters, each of which is further divided into two or three smaller sub-groups. Since compounds of different chemical classes evenly distributed in each of these clusters, we further examined the relative abundance of metabolites associated with known pathways of the central metabolism in order to search for common trends among functional groups.

In the pathways of carbohydrate and starch biosynthesis (Figure 2B, plots in light blue), the content of Suc increases as the florets approached anthesis, but progressively decreases soon after. Suc is synthesized in leaves and translocated to the floral calyx via the phloem. Alternatively, Suc can also be synthesized in flowers by the reaction catalyzed by sucrose synthase (SUS), which utilizes Fru-6P and UDP-Glc as substrates for Suc synthesis. As the content of sugar phosphates, Glc-6P and Fru-6P, sharply decreases in mature flowers, this is a possible indication that the flow through glycolysis and pentose phosphate pathways may slow down as the florets progress through anthesis. Conversely, the content of Fru increases sharply following the developmental progression from buds to mature flowers, as does trehalose of which the content doubles in open flowers versus buds just as Glc, although only in the last two stages of flower maturation. This suggests that the Suc imported into flowers is converted into monosaccharides and other carbohydrates which are presumably used for storage as flowers mature. Indeed maltose, which can be regarded as a proxy for starch, accumulates to high levels in the floral stage that immediately preceded anthesis (that is when flowers start secreting nectar) and to moderate levels when flowers transition to fruits. Minor sugars and sugar derivatives show peculiar trends. For example, the pools of *myo*-inositol and threitol decrease soon after anthesis, erythritol and trehalose increase, while galactinol and galactose maintain constant levels throughout development.

Many of the intermediates of the tricarboxylic acid (TCA) cycle (Figure 2B, plots in tangerine) and their glycolytic precursors, glycerate and pyruvate, display a short initial spike in young buds followed by a progressive decrease which stabilizes once flowers enter senescence. Citrate, isocitrate, pyroglutamate, and citramalate all show a progressive decrease in their content as flowers develop. However, the compounds that follow succinate in the progression of the TCA cycle, show a different trend. The level of succinate itself, for example, doubles in flowers at the stage of anthesis and then slowly falls to the initial level once flowers become older. This suggests that alternative pathways may feed metabolites into the TCA cycle. For example, the initial increase of succinate could be fueled by the degradation of γ-aminobutyric acid (GABA; Figure 2B, amino acids plots in green), of which the level sharply decreases up to the stage of anthesis and later increases again. The level of fumarate gradually increases and triplicates as flowers developmentally progress from young buds to a stage of mature organs, a trend which could be supported by the conversion of malate to fumarate by a cytosolic fumarase (Sweetlove et al., 2010).

The content of many amino acids increases or decreases during development, most probably in relation to their function as metabolic precursors of other compounds or depending upon their rate of import from the leaves (Figure 2B, plots in green). Ser, Gly, Thr, and Leu are all very abundant in postanthesis flowers, with Gly reaching a 20 times higher content in mature flowers than in buds. However, the level of these amino acids decreases again as flowers progress from 14.25 to 15.25 days of age. A very similar trend is also observed for amino acids derived from Asn (β-Ala, Lys, and Ile) and Gln (His, Pro, and Hyp) although their highest level is reached at around anthesis and decreases soon after. Asn and Gln are amino acids with a high N-to-C ratio, therefore preferentially used for long distance transport from source to sink tissues. Since the content of Asn and Gln reached its highest level at, or around anthesis, it can be speculated that the net transport of amino acids into flowers may slow down after this developmental point. Amino acids derived from the degradation of enzymes and proteins, which may not be further used as flower enter senescence, can also contribute to the high level of amino acids measured in mature flowers.

The aromatic amino acids Phe, Tyr, and Trp, as well as the intermediates and end-products of the shikimate pathway, are all abundant in flowers in postanthesis (Figure 2B, plots in lilac color). Conversely, the content of ascorbate, dehydroascorbate, and other minor compounds (Figure 2B, gray plots) displays a progressive decrease as flowers age.

#### Secondary metabolites

**Figure 3:**
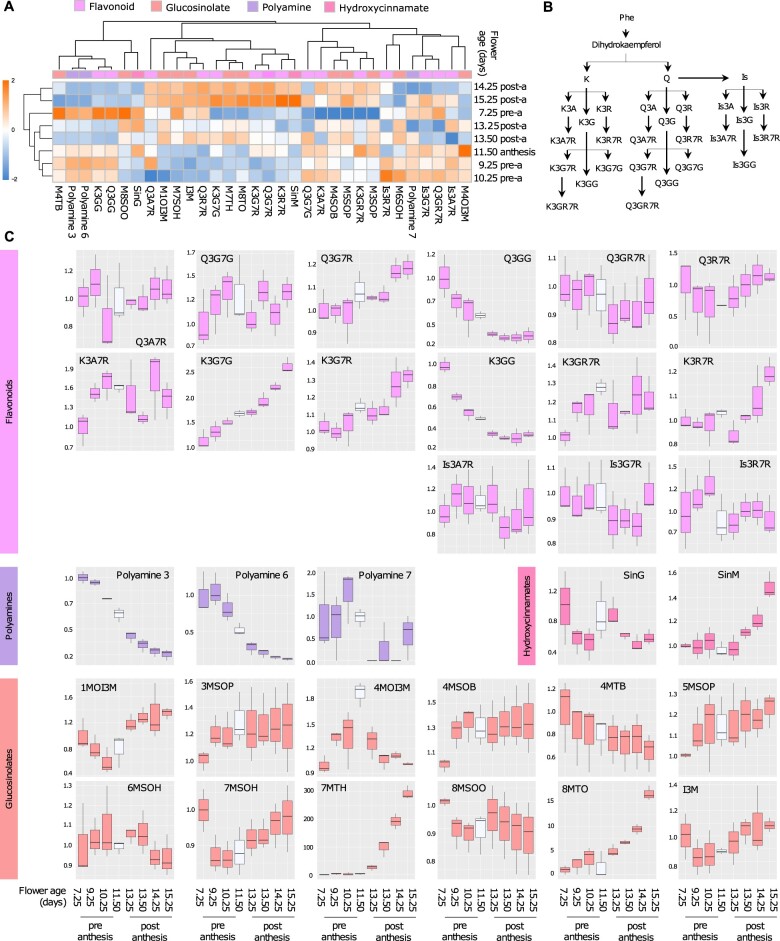
Profile of secondary metabolites in *A. thaliana* florets. A, Heatmap of secondary metabolites in eight stages of flower development. Florets in the stage of anthesis are 11.50 days old. Each square in the heatmap represents the log_2_ of the average metabolite content from three biological replicates each composed of ∼40 florets normalized by FW and internal standard. The colored bar atop the heatmap indicates the following classes of metabolites: lilac, flavonoid; purple, polyamines; pink, hydroxycinnamates; and rose peach, glucosinolates. B, Schematic representation of the pathway of flavonoid decorations. C, Boxplots representing the content of secondary metabolites measured in Arabidopsis florets in each of the eight stages of development relative to the youngest stage (7.29 days old buds). The boxplots show the median (central bar), the interquartile range (box), and minimum and maximum values (vertical bars). The color of the boxplots represents the classes of metabolites previously specified. A white plot represents florets in the stage of anthesis. A, arabinose; B, butyl; G, glucose; H, heptyl; I, indole; Is, isorhamnetin; K, kaempferol; M, methyl; O, octyl; P, propyl; Q, quercetin; R, rhamnose; S, sulfinyl; SinG, sinapoyl-glucose; SinM, sinapoyl-malate; and T, thio-substitution.

As we did for primary metabolites, we also annotated and quantified secondary metabolites (Supplemental Data Set S1). The heatmap of secondary metabolites of the class of flavonoids, glucosinolates, and polyamines shows two separate clusters. A first cluster which includes mature flowers of 14 days of age and older, and a second cluster with all the other developmental stages (Figure 3A). Within this second cluster, floral buds in the early stage of development (7.25 days old) form a separate group as they are characterized by very high levels of polyamines, quercetin-3-*O*-(-*O*-glucosyl)glucoside (Q3GG), and kaemferol-3-*O*-(-*O*-glucosyl)glucoside (K3GG), and very low levels of kaempferol-3-*O*-(2″-*O*-rhamnosyl)glucoside-7-*O*-rhamnoside, keampferol-3-*O*-arabinoside-7-*O*-rhamnoside, and numerous aliphatic glucosinolate compounds (Figure 3, A and C, plots in lilac; see also Figure 3B for a schematic representation of the main decorations of flavonoid compounds). The remaining samples in this second cluster form two small subgroups, one containing young buds and florets in the stage of anthesis (from 9.25 to 11.50 days old), and another group containing young open flowers (13.25 and 13.50 days old). In general, flavonoids obtained from the decoration of quercetin maintained fairly stable levels throughout flower development, with the exception of Q3GG, of which the level decreases along the developmental progression toward mature flowers, and quercetin-3-*O*-glucoside-7-*O*-rhamnoside which shows an opposite trend as its level increases in old flowers (Figure 3C, plots colored in lilac). The level of flavonoids obtained from the decoration of kaempferol mostly increases as flower aged as seen for K3G7G, K3G7R, and K3R7R with K3GG representing the sole exception as its amount decreases along with flower development. Flavonoids with the isorhamnetin backbone maintain stable levels during development. The levels of polyamine 3 and polyamine 6 (Figure 3B, plots in purple) decrease dramatically as flower aged, while the content of hydroxycinnamate sinapoyl-malate SinM increases (Figure 3C, plots in pink). Aliphatic glucosinolates 7-methylthioheptyl glucosinolate and 8-methylthiooctyl glucosinolate display a tremendous increase starting from the stage of anthesis, which highlights a possible developmental regulation of this class of compounds (Figure 3C, plots in rose peach). Although with a lower intensity, the content of the aliphatic glucosinolates 5-methylsulfinylpentyl and 7-methylsulfinylheptyl, and indole glucosinolates 1-methoxy-3-indolylmethyl and 4-methoxy-indol-3-ylmethyl (4MOI3M) also increase as flowers progressed through development. Interestingly, the level of 4MOI3M doubles at anthesis, and then quickly returns to the initial level.

#### Apocarotenoids, hormones, and terpenes

**Figure 4:**
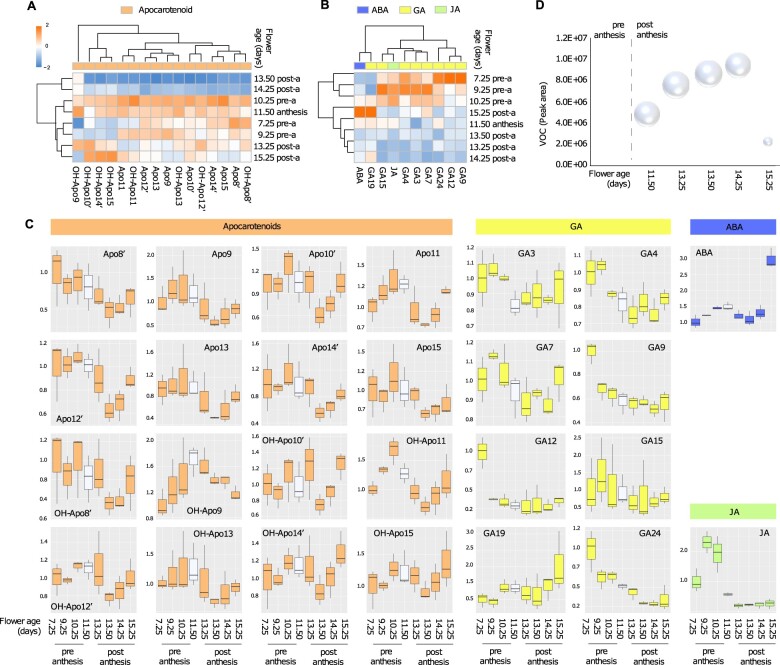
Profile of apocarotenoid, hormones, and VOCs in *A thaliana* florets. A, Heatmap of apocarotenoids measured in eight stages of flower development. Florets in the stage of anthesis are 11.50 days old. Each square in the heatmap represents the log_2_ average of metabolite content from three biological replicates each composed of ∼40 florets normalized by FW and internal standard. The colored bar atop the heatmap indicates the following classes of metabolites: orange, apocarotenoids. B, Heatmap of hormones measured as described in (A). The colored bar atop the heatmap indicates the following classes of metabolites: blue, ABA; yellow, GA; lime green, JA. C, Boxplots representing the content of apocarotenoids and hormones measured in Arabidopsis florets in each of the eight stages of development relative to the youngest stage (7.29-day-old buds). The boxplots show the median (central bar), the interquartile range (box), and minimum and maximum values (vertical bars). The color of the boxplots represents the classes of metabolites previously specified. A white plot represents florets in the stage of anthesis. D, Emission of VOCs of the class of terpenoids from 40 individual florets detached from the main inflorescence collected in an open loop system. The size of the beads represents the total peak area of terpenoid VOCs measured at each stage of flower development. Apo, apocarotenoid.

Based on the content of apocarotenoids (Supplemental Data Set S1), flower samples cluster into two well defined clusters visible on a heatmap (Figure 4A). The first cluster includes open flowers in the stages between postanthesis and senescence (13.50 and 14.25 days old), with very low apocarotenoids content. The second cluster includes buds and open flowers in all the other stages of development, which accumulate apocarotenoids in variable amounts. In this second cluster, the largest number of compounds is produced by flowers approaching anthesis or in the stage of anthesis. However, the production of apocarotenoids is fairly stable throughout flower development, as seen from the plots showing the relative apocarotenoid abundance (Figure 4C, plots in orange). Only a few apocarotenoid compounds, namely OH-Apo-10′, OH-Apo-14′, and OH-Apo-15, show relatively high content in senescing flowers.

Flower development is temporally coordinated by the activity of plant growth regulators of the class of GA, JA, and abscisic acid (ABA) (Shan et al., 2019). The heatmap in Figure 4B shows elevated JA and GA levels in bud samples, which indeed form a separate cluster on the heatmap. A second cluster, which groups all open flower from anthesis to maturity, is characterized by a very low content of plant growth regulators. Within this group, 15.25 days old senescing flowers are the only exception with respect to the content of GA19 and GA7 (see also Figure 4C plots in yellow) and ABA, of which the content triples in old senescing flowers (see also Figure 4C, plots in blue).

Arabidopsis flowers emit a small bouquet of volatile terpenoid compounds synthetized by two sesquiterpene and two monoterpene synthases (Chen et al., 2003; Tholl et al., 2005). The quantification of this small group of VOCs revealed increased emission starting in flowers in the stage of anthesis and proceeding until maturity, after which the emission of VOCs abruptly comes to a halt (Figure 4D).

#### Combinatorial analysis of metabolites

**Figure 5:**
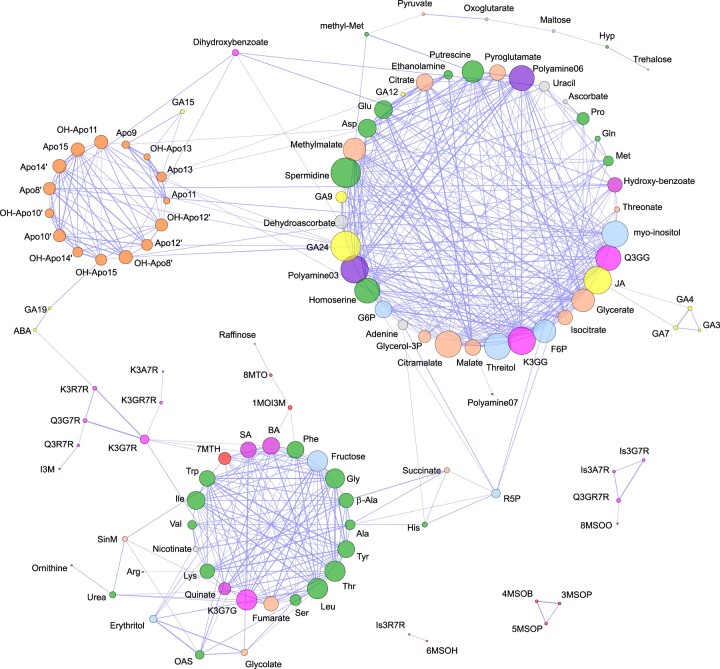
Correlation network of Arabidopsis floral metabolites. An organic layout in Cytoscape was chosen to visualize the correlation between metabolites (Pearson correlation threshold of 0.65). Nodes represent metabolites and edges and the interaction between metabolites. The size of nodes and edges maps to degree and correlation coefficient, respectively. Different classes of metabolites are represented with different colors: amino acids, green; organic acids, tan; carbohydrates, light blue; hormones, yellow; apocarotenoids, orange; flavonoids, pink; polyamine, purple; glucosinolates, red; other, gray.

We investigated the relationships between different classes of metabolites via Pearson pairwise metabolite to metabolite correlations (Supplemental Data Set S2). Out of the 8,257 possible correlations, 2,679 pairwise comparisons were significant (*P* ≤ 0.05), and of these, 509 showed strong positive (*r^2^* > 0.65) and 335 negative (*r^2^* < −0.65) correlation coefficients. To facilitate data interpretation, the high positive correlations were chosen to compute a network (Figure 5), while the whole correlation dataset was visualized on a heatmap where metabolites were grouped by compound class (Supplemental Figure S1). The resulting network shows three clusters of highly correlated metabolites. The largest cluster is very heterogenous as it includes metabolites belonging to very different chemical classes. Here, highly connected and relevant nodes are represented by plant growth regulators GA24 and JA, organic acids glycerate, malate, citramalate, and methylmalate, sugar alcohols myo-inositol and threitol, and sugar phosphates Glc-6P and Fru-6P. A second large cluster, which is more homogenous in its constituents, includes aliphatic and aromatic amino acids, and components of the shikimate pathway and derivatives. A third highly homogeneous cluster only includes apocarotenoids. Overall, cross-cluster correlations were sparse and mostly limited to correlations between GA24 and apocarotenoids. Relatively large clusters of negative correlations were found between amino acids and apocarotenoids which are visible on the heatmap (Supplemental Figure S1).

### Isotope labeling kinetics in whole inflorescences and single florets

We next asked the question how do flowers support such a great level of metabolic diversity in terms of energetic requirements and the need for various metabolic precursors, given that, in flowers, only sepals of young floral buds carry out photosynthesis. Indeed, for most of their lifespan, flowers are essentially heterotrophic and primarily sustained by the Suc which reaches the floral calyx via the source-to-sink translocation system of the phloem. It is known that at the floral calyx, cell wall invertases (cwINVs) unload sucrose from the phloem by breaking it down to Glc and Fru (Ruan, 2022), and then Glc is mainly transported into the floral tissues because of the predominant presence of numerous transporters and carriers facilitating its fast uptake (Borghi and Fernie, 2017). However, it is not yet known to what extent Glc is partitioned and utilized for the synthesis of soluble molecules, such as carbohydrates, organic acids, and amino acids, as well as large insoluble polymers like starch and cell wall components. The differential contribution of glycolysis and respiration in supporting the energetic requirements of flowers is also poorly investigated. Finally, it is not well understood which of the numerous pathways composing the network of the floral primary metabolism is preferentially targeted for Glu channeling and utilization, and whether the direction of this metabolic flux differs in preanthesis versus postanthesis. To assess the metabolic fate of Glc in flowers, we performed flux analyses upon feeding radio-labeled [U-^14^C]-Glc and stable isotope-labeled [U-^13^C]-Glc to whole inflorescences.

In a first labeling experiment, in order to broadly characterize the major fluxes in flowers, we measured the incorporation of radiolabeled carbon into the major classes of chemical compounds after fractionation by ion-exchange chromatography coupled with enzymatic digestion of whole inflorescences fed with [U-^14^C]-Glc (Obata et al., 2017). The outcome of this experiment revealed a predominant redistribution of the radiolabeled carbon into the pool of hexose phosphates and a dominant flux toward the synthesis of Suc (Table 1). These results align well with the model of Suc breakdown and re-synthesis, also known as a “futile cycle” which flowers utilize to move carbohydrate resources into different floral tissues bypassing the apoplasmic barriers which separate individual floral organs within flowers. The low evolution of CO_2_ which we measured in this experiment, also suggests that glycolysis and the pentose phosphate pathways may be the preferential routes for the breakdown of Glc in flowers.

**Table 1:** Redistribution of radiolabeled [U-^14^C]-Glucose and fluxes in whole inflorescences of *A. thaliana*

Measured variable	Mean ± sd
Label incorporated (Bq gFW^−1^)	
Total uptake	1.36 ×10^7^ ± 3.89 × 10^6^
Metabolized radioactivity	1.29 × 10^7^ ± 3.72 × 10^6^
Recovery	0.58 ± 0.04
Redistribution of radiolabel carbon (% of total metabolized)	
CO_2_	0.10 ± 0.03
Amino acids	0.39 ± 0.06
Organic acids	35.29 ± 3.79
Hexoses-P pool	53.24 ± 5.12
Suc	6.42 ± 0.95
Fru	1.59 ± 1.19
Protein	0.57 ± 0.14
Starch	0.17 ± 0.03
Cellulose	2.23 ± 0.32
Metabolic flux (nmol hexose equivalents gFW h^−1^)	
Suc synthesis	2.57 × 10^4^ ± 1.28 × 10^4^
Starch synthesis	745.17 ± 499.09
Cellulose (cell wall) synthesis	9167.26 ± 5049.41
Protein synthesis	2466.28 ± 1725.3

Inflorescences were cut from the main bolting stem of flowering Arabidopsis plants and incubated in a solution of 100 mM K_2_SO_4_ and 100 mM Glc (specific activity 7MBq mmol^−1^ of total glucose) for 5 h. At the end of the incubation period, whole inflorescences were newly cut from the stems that remained in direct contact with the feeding solution, extracted, and analyzed for radiolabel in amino and organic acids, starch, proteins, cell wall, phosphoesters, and sucrose. ^14^CO_2_ evolved from flowers was trapped in KOH and the level of radioactivity determined by scintillation counting. Absolute rates of flux were calculated from the label incorporation data using the specific activity of the hexose-P pool to account for isotopic dilution factors. Values are means ± sd (*n* = 5 samples each composed of three inflorescences).

In a second labeling experiment, we fed [U-^13^C]-Glc to whole inflorescences from which we harvested individual florets in the stages of preanthesis, anthesis, and postanthesis. We extracted metabolites from these samples and quantified the total incorporation of ^13^C in the primary metabolites of the central pathway, after correcting for the natural abundance of ^13^C (Table 2). The results of this experiment show that the incorporation of ^13^C into the pool of amino acids peaks in preanthesis and decreases as flowers advance their phenology from preanthesis to anthesis and postanthesis. The pool of the organic acid intermediates of the TCA cycle displays a maximum of ^13^C incorporation at anthesis. Similarly, ^13^C labeling of the pool of carbohydrates also reaches its highest point at anthesis, although it retains an elevated level also in postanthesis. Therefore, ^13^C labeling reveals that within the inflorescence, individual florets in different stages of development differentially allocate resources toward direct synthesis of amino acids, TCA intermediates, and carbohydrates. Metabolites with a bioenergetic function, such as TCA intermediates and carbohydrates, show a maximum peak of ^13^C incorporation starting in preanthesis, proceeding during anthesis and which also extends to postanthesis. Novel synthesis of amino acids peaks in preanthesis and may also continue across anthesis as the total content of many amino acids, which includes labeled and unlabeled amino acids, reaches a peak in this developmental stage (Supplemental Table S1). Moreover, amino acids which derive from protein degradation, and which are unlabeled due to the short time of labeling, may also contribute to this pool. Overall, ^13^C labeling shows that the stage of anthesis is a moment of intense metabolic activity in the life of a flower, characterized by high rates of anaerobic and aerobic respiration and recycling of metabolites.

**Table 2:** Label accumulation in individual florets following [U-^13^C]-Glc feeding of whole *A. thaliana* inflorescences

		Stage of development											
Metabolite	Group	Preanthesis		Anthesis		Postanthesis							
		Avg		se		Avg		se		Avg		se	
Ala	Amino acid	328.92	±	47.59	a	466.36	±	45.78	b	414.00	±	30.45	b
Asn	Amino acid	155.95	±	20.95	a	125.28	±	22.20	a	50.20	±	6.60	b
Asp	Amino acid	326.42	±	41.04	a	152.66	±	14.77	b	77.69	±	3.83	c
GABA	Amino acid	46.58	±	6.64	a	37.20	±	5.02	a	26.46	±	6.11	b
Glu	Amino acid	2670.82	±	297.74	a	1714.00	±	172.21	b	799.73	±	85.65	c
**Glycine**	**Amino acid**	**101.92**	**±**	**6.08**	**a**	**83.07**	**±**	**1.08**	**b**	**49.86**	**±**	**5.64**	**c**
Ile	Amino acid	3.02	±	0.13	a	2.93	±	0.71	ab	2.17	±	0.14	b
Leu	Amino acid	1.52	±	0.20	a	1.08	±	0.30	ab	0.59	±	0.13	b
Ornithine	Amino acid	4.43	±	0.71	a	3.15	±	0.42	b	2.85	±	0.33	b
Pro	Amino acid	333.88	±	53.28	a	274.55	±	13.97	a	121.66	±	8.61	b
Ser	Amino acid	210.43	±	28.11	a	141.64	±	14.54	b	79.35	±	8.83	c
Thr	Amino acid	51.85	±	7.17	a	60.39	±	5.89	a	45.45	±	6.14	a
**Val**	**Amino acid**	**178.44**	**±**	**15.81**	**a**	**139.77**	**±**	**3.70**	**b**	**58.98**	**±**	**6.36**	**c**
**Fru**	**Carbohydrate**	**437.16**	**±**	**103.16**	**a**	**2378.80**	**±**	**25.09**	**b**	**2418.67**	**±**	**212.58**	**b**
**Glc**	**Carbohydrate**	**1.71x10^5^**	**±**	**1.47x10^4^**	**a**	**4.84x10^5^**	**±**	**1.71x10^4^**	**b**	**3.74x10^5^**	**±**	**4.62x10^4^**	**c**
**Myoinositol**	**Carbohydrate**	**627.30**	**±**	**95.75**	**a**	**570.21**	**±**	**6.39**	**a**	**364.14**	**±**	**21.13**	**b**
Suc	Carbohydrates	10576.81	±	1571.98	a	12052.37	±	421.79	a	6878.57	±	391.95	b
Trehalose	Carbohydrates	17.92	±	1.57	a	13.29	±	4.38	a	26.59	±	0.78	b
**Citrate**	**Organic acid**	**588.81**	**±**	**87.27**	**a**	**276.14**	**±**	**0.04**	**b**	**436.99**	**±**	**8.60**	**c**
**Fumarate**	**Organic acid**	**69.86**	**±**	**6.24**	**a**	**316.96**	**±**	**33.09**	**b**	**220.42**	**±**	**9.16**	**c**
Glycerate	Organic acid	11.27	±	4.60	a	23.93	±	4.09	b	15.18	±	5.73	ab
Malate	Organic acid	498.35	±	48.09	a	654.58	±	31.01	b	364.05	±	26.89	c
Succinate	Organic acid	137.83	±	14.18	a	431.74	±	27.73	b	155.27	±	6.66	a

Inflorescences were cut from the main bolting stem of flowering plants and incubated in a solution of 100 mM K_2_SO_4_ and 100 mM [U-^13^C]-Glc for 5 h. At the end of the incubation period, florets in the stage of preanthesis, anthesis, and postanthesis were separately harvested, metabolites extracted, and the total incorporation of ^13^C in each metabolite quantified. Values represent the average ^13^C accumulation (ng· mg^−1^ FW) per metabolite (*n* = 3 samples ± se). Different letters indicate values significantly different at *P* *<* 0.05, while metabolites in bold are significantly different at *P* *<* 0.01 in at least one of three possible pairwise comparisons (*t* test).

### Genes associated with the metabolism of carbohydrates, and transport of carbohydrates and amino acids are highly represented at the transition from close to open flowers

To gain further insights into the molecular processes underlying the metabolic shift occurring at the onset of flower anthesis, we extracted RNA from pools of ∼40 individual florets in each of the eight previously described stages of development, and quantified transcript abundance via RNA sequencing. A total of 29,000 transcripts were initially identified and from which we excluded from further analyses genes with less than ten counts and genes that did not show significant changes in at least one of the possible contrasts between developmental stages. We also excluded transcripts that did not fit a quadratic regression (df = 3), as changes in transcript abundance across development are expected to be smooth and gradual. The multi-dimensional plot of the 19,388 normalized counts that remained after filtering shows a clear segregation of flower developmental stages and therefore highlights existing differences among the samples and the robustness of the dataset (Supplemental Figure S2 and Supplemental Data Set S3). Comparison of transcript abundance across developmental stages showed that the largest number of significantly differentially expressed genes (DEGs) belongs to the early transition between 9.25 and 10.25 day old buds, after which the number of DEGs remained stable until 13.50 day old flowers, and it started to decrease soon after (Supplemental Data Set S4). Gene ontology (GO) enrichment analysis revealed enrichment of terms related to catalytic activity, ion binding, and transmembrane transport activity (GO category “molecular function”), cell periphery and plasma membrane (GO category “cellular component”), and response to stimulus (GO category “biological process”; Supplemental Data Set S4 and Supplemental Figure S3). Term enrichment analysis performed at the Kyoto Encyclopedia of Genes and Genomes (KEGG) revealed enrichment of genes associated with the metabolic pathways of glycolysis, TCA cycle, and sucrose metabolism, as well as glucosinolate and flavonoid biosynthesis the latter two occurring at the developmental transition between bud and anthesis and between anthesis and mature flowers, respectively (Supplemental Figure S4). The comparison of DEGs in the three major developmental groups of preanthesis, anthesis, and postanthesis revealed high transcriptional activity of genes associated with the photosynthetic process, as well as enrichment of KEGG terms associated with porphyrin and chlorophyll metabolism in buds in preanthesis (Supplemental Figure S5, A and B). In mature postanthesis flower, genes of the categories of sugar and hormone signaling and transport are instead highly prominent (Supplemental Figure S5, A and C). The phase of flower opening is characterized by a small group of 369 upregulated genes, among which DEGs associated with the terms “endomembrane system” and “Golgi,” as well as genes involved in the biosynthesis of glucosinolates are the most abundant (Supplemental Figure S5A).

**Figure 6:**
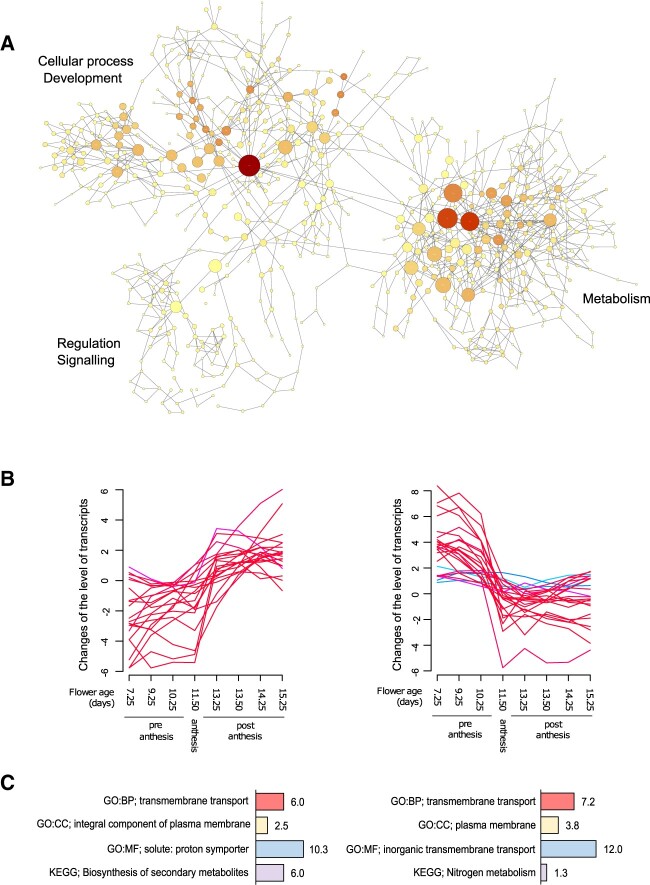
Visualization of transcript classes overrepresented across flower development. A, Network visualization of the overrepresented GO categories in the correlation network of selected transcripts across all flower developmental stages (correlation cutoff 0.82). Size and color of nodes are proportional to their relevance in the network with bigger and darker nodes showing overrepresented GO categories. B, Clusters of transcripts showing opposite behavior at the onset of anthesis. C, GO term enrichment of transcripts represented in (B). Bars show the negative log_10_ of the adjusted *P* value of the main enriched term in the GO categories biological process (red), cellular components (yellow), molecular function (blue), and KEGG (purple).

Correlative associations across transcripts were investigated on the subset of the first 9,000 DEG which emerged as significant across all developmental comparisons. The resulting 655,666 positive correlations (correlation cutoff 0.82; unique genes 7,572) were plotted on a network and analyzed from the perspective of functional grouping with the BiNGO tool in Cytoscape (Maere et al., 2005). In the resulting network of 783 nodes and 1,347 edges, we identified two clusters of highly correlated nodes and a smaller cluster of more loose connections (Figure 6A andSupplemental File S1). The first cluster of highly correlated nodes is enriched in genes associated with cellular processes regulating development of reproductive structures, including fruits, seeds, and embryo development. Here, the GO term descriptors associated with vesicle-mediated transport from the ER to the Golgi apparatus, retrograde transport between endosomes to Golgi (the cellular process that recycles membrane proteins during growth and development), cellular membrane organization, organization of chloroplast, and peroxisomes were all enriched (Supplemental Data Set S5; Cluster Development), which highlight the membrane trafficking processes contributing to meristem growth and development. The second cluster of highly connected nodes is enriched in the GO descriptors associated with metabolism. Here, the bigger and brighter nodes which represent the most relevant nodes in the network include the terms cellular metabolic process and primary metabolic process and the descendant terms carbohydrate, amino acid, protein, and lipid metabolic processes indicating the great involvement of metabolites in the developmental process that spans across anthesis (Supplemental Data Set S5; Cluster Metabolism). Direct correlation between the central nodes of the network subclusters “development” and “metabolism” clearly points at the interdependence between these two processes. Finally, a third smaller cluster includes positive and negative regulatory genes of development and metabolism (Supplemental Data Set S5; Cluster Signaling).

We next focused attention on the steady-state level of transcripts of genes with experimental and computational evidence of association with known metabolic pathways of the central and secondary metabolism (Schlapfer et al., 2017). K-mean clustering applied to this subset of genes unraveled two hidden clusters characterized by sudden and opposite changes of transcript levels occurring at the onset of anthesis (Figure 6B). GO term enrichment revealed that genes in both clusters associate with transmembrane transport of organic and inorganic solutes, such as ion, peptides, organic acids, and sugars (Figure 6C). *SUGAR TRANSPRTER PROTEIN 9* (*STP9*), *STP6*, *POLYOL/MONOSACCHARIDE TRANSPORTER 2*, and *SUCROSE WILL EVENTUALLY BE EXPORTED TRANSPORTER 5* (*SWEET5*) belong to the cluster of genes of which the level of transcripts increases at the onset of anthesis. The genes *HEXOKINASE 3*, *PYRUVATE KINASE COMPLEX*, *MALATE SYNTHASE*, and *MALATE DEHYDROGENASE*, and genes of the Major facilitator superfamily proteins, which enable carbohydrate membrane transporter activity, were also represented in this cluster. In the second cluster which includes genes with negative changes in the level of transcripts at the onset of anthesis, we observed the nitrate and nitrate excretion transporters *NRT1.6*, *NRT2.6*, *NRT3.1*, and *NAXT1* and the amino acid permease *AAP8*. The *SUCROSE PROTON SYMPORTERS 7* (*SUC7*), *SUC9*, *SUS4*, and a few genes of the major facilitator superfamily proteins are also present in this cluster, which suggest that a different subset of genes of the sugar metabolism are activated as flowers open. Genes associated with the conversion and production of energy such as photosynthesis (Supplemental Figure S6, A and B), glycolysis (Supplemental Figure S6E), and the TCA cycle (Supplemental Figure S6H) also accumulate in high abundance in flower in preanthesis of which the abundance of transcripts decreases as flowers age. Given that flowers are described as sink tissues, it was initially unexpected to see high level of transcripts associated with the Bassham–Benson–Calvin cycle occurring all throughout anthesis. However, in Arabidopsis embryos chlorophylls accumulate in the cotyledons early during development. Moreover, the siliques themselves are photosynthetically active and most probably capable of providing sufficient carbohydrate resources to reinforce glycolysis and TCA cycle (Brazel and O’Maoileidigh, 2019). Transcripts associated with the shikimate pathway (Supplemental Figure S6J), branched chain amino acids (Supplemental Figure S6K), carotenoid (Supplemental Figure S6I), and ABA (Supplemental Figure S6O) biosynthesis only accumulate in marginal amounts during anthesis.

### Transcript to metabolite correlations

**Figure 7:**
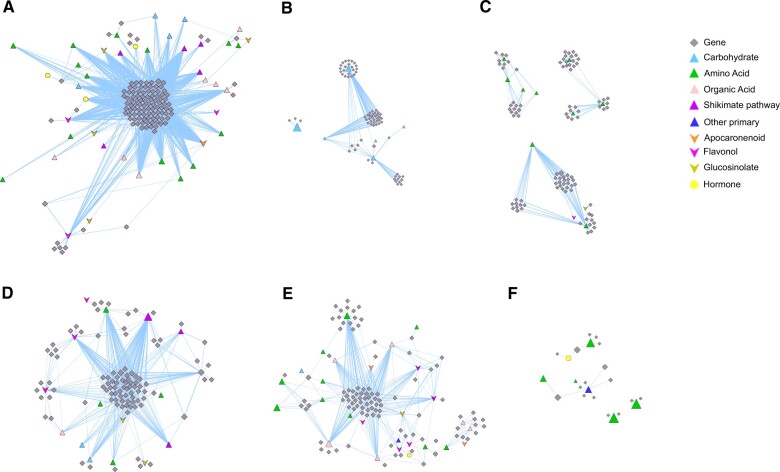
Gene-metabolite correlation networks. A, Visualization of the Pearson correlations between transcripts and metabolites in Arabidopsis florets. A–C, developmental stages from S9 to S13 (preanthesis) and (D)–(F), developmental stages from S13 to S16 (postanthesis). Genes are represented with gray diamond symbols, primary metabolites with triangles, secondary metabolites with inverted triangles, and hormones with circles. Carbohydrates are colored in light blue, amino acids in green, organic acids in pink, metabolites of the shikimate pathway in purple, other primary metabolites in deep blue, apocarotenoids in orange, glucosinolates in cress-green, and flavanols, hydroxycinnamates, and polyamines with different shades of purple-violet. The correlation matrix was computed using the R package Hmisc and represented with an organic layout in Cytoscape version 3.8.2. Spring length and mass was manually adjusted to avoid node overlapping. The size of nodes is proportional to the network parameter of betweenness centrality, with larger nodes representing highly connected metabolites and genes in the network. Edge thickness is proportional to the correlation value with thicker edges representing stronger correlations. Only positive correlations above the value of 0.80 are represented.

From groups of florets in preanthesis and postanthesis, we calculated Pearson correlations between metabolites and transcripts of genes with experimental or putative association with known metabolic pathways, and ultimately plotted the positive correlations (*r*^2^ > 0.80) on two separate networks (Supplemental Figure S7 and Supplemental Files S2 and S3). The network representing flowers in preanthesis (Supplemental Figure S7A), shows a large cluster of highly connected genes and metabolites and a series of smaller clusters more loosely connected with one another. The larger cluster (Figure 7A) includes numerous genes of the family of amino acids, peptide, and nitrate transporters, including general transporters able to bind different combinations of amino acids such as *ARABIDOPSIS THALIANA PEPTIDE TRANSPORTER 1* (*ATPT1*) and *ATPT3*, *PROLINE TRANSPORTER*, and members of the major facilitator superfamily which may help to retrieve nitrogen from the xylem and mobilize it to the whole flower or specific floral tissues. Numerous sucrose transporters are also represented in this large cluster which include genes of the *SWEET* family (*SWEET1*, *SWEET4*, *SWEET13*, and *SWEET14*), *INOSITOL TRANSPORTER 2*, *SUCROSE CARRIER* (*SUC2*), and the related *SUS1*, *SUS3*, and *SUS5*. Nodes representing metabolites in this large cluster are the amino acids Pro and hydroxyproline (Hyp), the aliphatic amino acids Gly, Ala, β-Ala, and Ile, and the aromatics Tyr and Trp. Putrescine and urea also correlate with this large cluster of genes. Among the carbohydrates, we noticed the presence of Fru, trehalose, galactose, erythritol, and maltose suggesting their transport from tissue to tissue as organs of flowers develop. Moreover, starch degradation is also a signature of florets in preanthesis as the presence of *PHOSPHOGLUCOMUTASE* and *GLUCOKINASE* genes also suggest. We also noticed many genes with putative localization to anthers and pollen, highlighting the presumably large dependence of these tissues on the transport of primary metabolites of the class of amino acids and sugars. Genes involved in sucrose and starch metabolism, glycolysis, decarboxylation of pyruvate, and ultimately the TCA cycle all point toward the needs of flowers in preanthesis for high energy level metabolism. The remaining more loosely connected and smaller clusters (Figure 7, B and C) show the sugar phosphates (Ribulose-5P, Glc-6P, and Fru-6P) and galactinol as highly influential nodes in the pathway being characterized by high values of betweenness centrality (Supplemental File S2). While here many genes associated with transport and metabolism of sugars and amino acids are also clustering together, it is also interesting to recognize the presence of genes of the Bassham–Benson–Calvin cycle, as well as genes of the pentose phosphate pathway which has been shown to provide biosynthetic precursors for the synthesis of nucleic acids essential for embryo development (Andriotis and Smith, 2019). GABA and numerous classes of GA (GA4, GA9, GA12, and GA24) are connected to genes of the geranylgeranyl diphosphate biosynthesis (MEP pathway). Polyamines and derivatives of kaempferol and quercetin (Q3GR7R, K3GG, and Q3GG) also appear in this part of the network.

The network pertaining to flowers in postanthesis (Supplemental Figure S7B and Supplemental File S3) shows two major clusters of transcript-to-metabolite correlations and a few metabolites and genes with high values of network connectivity. The larger cluster is predominantly characterized by amino acids, small peptide, and nitrate transporters some of which still lack functional characterization in floral tissues or in planta (Figure 7D). Here, we see genes of the major facilitator superfamily proteins, *AMINO ACID PERMEASE 1* (*AAP1*), *AAP3*, and *AAP6*, *NITRATE TRANSPORTER 1* (*NRT1*), *PEPTIDE TRANSPORTER 1*, and *LYS-HIS-LIKE TRANSPORTERS).* Many genes of the arogenate and chorismite pathway (aromatic amino acids) are also represented in the subcluster. Relevant nodes in this cluster include the metabolites benzoic (BA) and salicylic acids, Phe, and derivatives of kaempferol (K3G7G) and quercetin (Q3R7R), and a few hydroxycinnamates and glucosinolates. The other large cluster (Figure 7E), shows a few amino acid transporters and genes associated with carbohydrate metabolism such as *cwINV4*, *HXK1*, and *SWEET* transporters (*SWEET4*, *SWEET13*, and *SWEET14*). All the genes in this large cluster are highly connected with key metabolites of the TCA cycle (citrate, isocitrate, succinate, malate, and methyl-malate) and pyroglutamate. Small nodes representing metabolites in the cluster also include kaempferol and quercetin derivatives. Nodes showing high level of network connectivity in this clusters represent the hormone ABA and the amino acids Hyp, Thr, ethanolamine, Ala, β-Ala, and urea. These nodes are strongly connected with the nitrate transporters *NPF2* and NRT3, and the pyrophosphate-dependent phosphofructokinase B subunit (*MATERNAL EFFECT EMBRYO ARREST 51*) which has been shown to promote embryo development (Figure 7F).

## Discussion

Flower metabolism has been the subject of numerous studies. However, research in this field primarily centered attention on a discrete number of metabolite classes, such as scent and color, and less frequently on multiple types of metabolites or the entire set of compounds that flowers synthesize, which is referred to as the metabolome. As flowers integrate signals from the whole plant to successfully channel metabolism toward pollination and reproduction, taking a metabolomic approach to the study of flower metabolism holds the promise to provide a thorough understanding of how flowers function as whole entities. Here, we provided a holistic overview of the entire set of primary and secondary metabolites and hormones of flower samples, which we further integrated with transcriptomics and radiolabeled feeding experiments. We focused attention on flower anthesis, as in this particular developmental transition pollination occurs, and the floral metabolome, initially programmed to support pollination, must be re-programmed to support seed and fruit set. We showed that primary metabolism plays a central role in this developmental transition, and changes at both transcript and metabolite levels follow the developmental progression of individual organs. The dataset presented here could be used to validate, confute or formulate new hypotheses pertaining to the role of individual metabolites or metabolite classes in regulating flower development. Additionally, we envision that this dataset could also be utilized to introgress relevant floral metabolic traits to promote pollination in crops species for which a comprehensive knowledge of flower metabolism is still limited.

### Anthesis is accompanied by a developmentally regulated reshaping of flower primary metabolism

Our survey of the Arabidopsis floral metabolome shows that the levels of metabolites of the central pathway change consistently during the transition from close to open flowers as to follow floral development from the stage of buds, across anthesis, up to mature and senescing flowers (Figure 2, A and B). Metabolites highly represented in the stage of preanthesis are sugars phosphates (G6P and F6P), maltose and sucrose, and intermediates of the TCA cycle (pyruvate, citrate, and malate). Comprehensively these data suggest that an intense metabolic activity takes place in preparation of flower opening. In *N.attenuata*, where the content of primary metabolites in the floral limb was measured before and after corolla opening, elevated sugars and TCA cycle intermediates were also detected (Stitz et al., 2014). Here, the prominent role of sugars was attributed to regulating cell turgor and expansion. Conversely, in petals of snapdragon where the water content increases only by 5% as flowers open, elevated sugars may instead support the increase in biomass via new cellulose synthesis and cell wall deposition (Muhlemann et al., 2014). Arabidopsis flowers between stages S9 and S13, the time frame during which we surveyed elevated levels of sugars and TCA intermediates, are certainly characterized by the appearance and growth of petal primordia, but also by the initial stages of stamen development up to the formation of the anther loculi (Smyth et al., 1990). Anthers are strong sinks of Suc and carbohydrates, and within the anthers the tapetum is a tissue characterized by a very intense metabolic activity. Here, sugars are exported to the locular fluid which nourishes the growing pollen grains (Pacini et al., 1985) while the fast turnover of the TCA cycle provides high energy intermediates (Busi et al., 2011). In this stage, specific classes of amino acids, as for example Pro, are also delivered to the microsporocytes. Indeed, [U-^13^C]-Glc feeding experiments showed elevated incorporation of label into Pro reaching a maximum in preanthesis and anthesis (Table 2). The pistil also differentiates at around this time, as the stigmatic papillae must be fully receptive as flowers open, and while transitory starch accumulates at the base of the floral calyx well before flower opening, at anthesis starch accumulation appears in the parenchyma cells of the stigma (Hedhly et al., 2016). Our dataset revealed that the content of maltose, which is obtained from the degradation of starch to amylopectin, is very high in 10.25 day-old florets, the time point immediately preceding anthesis.

Bellaire et al. (2014) measured elevated levels of amino acids with high N:C ratio in the early stages of flower development, for which they hypothesized that a conspicuous assimilation of nitrogen takes place in Arabidopsis florets in preanthesis. The results of [U-^13^C]-Glc feeding experiment validate this hypothesis (Table 2) and additionally show that label incorporation into specific carbohydrates and TCA intermediates rises at anthesis and is maintained at high level also in postanthesis (e.g. Suc and citrate). Here, differences in the level of specific metabolites are conceivably traceable to the use by Bellaire and coauthors of an inducible mutant line with a cauliflower-like habitus, in which the source–sink relationships may not be representative of a wild-type inflorescence. Indeed, our [U-^14^C]-Glc feeding experiment shows that in whole inflorescences, where all the progressive stages of development from flower primordia to senescing flowers are present, the primary sink of label incorporation are hexose phosphates and Suc (Table 1).

As flowers transition to postanthesis, primary metabolism does not show signs of slow quenching. Instead, a rather abrupt shift in the content of primary metabolites draws a dividing line between anthesis and postanthesis (Figure 2A). Here, sugars such as Fru and R5P are present in large abundance, as well as maltose, galactose, and trehalose which are present at high concentration especially in senescing flowers transitioning into fruits (15.25 day-old florets). At the time of pollination, sugars in stigma and style are mobilized to support the growth of the pollen tube while it elongates toward the ovule (Goetz et al., 2017). Following fertilization, carbohydrates still accumulate in early developing embryos, despite Arabidopsis seeds, as it is for other Brassicaceae species, mostly accumulate lipids and amino acids. Initially, starch accumulates in the endosperm, after which it is gradually degraded (Hedhly et al., 2016). Indeed, in our experiment, we measured high content of maltose (the disaccharide immediately derived from starch degradation) in three critical time points (Figure 2A): in preanthesis, and in postanthesis at the time of postfertilization (13.50 day-old florets) and the heart stage of embryo development (15.25 day-old florets; Stage S16 ; Figure 1). For the embryo to developmentally progress beyond the globular stage, the contribution of primary metabolic pathways providing R5P for the biosynthesis of purines, His, and NADPH is deemed to be indispensable. In fact, mutants of the oxidative pentose phosphate pathways and the G6P/phosphate translocator *gpt1* are embryo lethal as they prematurely cease to develop when they reach the globular stage (Andriotis et al., 2010; Andriotis and Smith, 2019). Additionally, the total content of numerous labeled and unlabeled amino acids is particularly elevated in postanthesis (Supplemental Table S1), as well as the level of transcripts of amino acid and peptide transporters (Figures 6, B and C and 7B and Supplemental Figure S6). As the incorporation of ^13^C labeling into the pool of amino acids peaks at the stages of preanthesis and anthesis, our finding also supports the hypothesis of Gaufichon et al. (2017) that amino acid reserves which are generated and stored in preanthesis are mobilized after pollination to support embryo development up to the heart stage.

### Secondary metabolites display a discontinuous distribution across anthesis

While primary metabolism marks the whole process of flower opening with high levels of compounds and transcripts being present at each of the eight time points we sampled florets, conversely, secondary metabolites show a discontinuous distribution. We observed considerable variation across different metabolites of the class of flavonoids irrespective of their chemical origin whether they are kaempferol, quercetin, or isorhamnetin derivatives (Figure 3). Our observations made in Arabidopsis flowers differs from studies performed in distantly related plant species, such as Fresia, where the concentration of multiple kaempferol derivatives was seen to increase up to anthesis (Shan et al., 2020). In fact, in Arabidopsis, K3G7G, K3KG7R, and K3R7R progressively accumulate starting from young buds, but their content continues to rise after fertilization. Moreover, among the kaempferol derivatives, K3GG displayed an opposite trend characterized by a remarkable decline in intensity from buds to open flowers. Nevertheless, all throughout flower opening the content of numerous flavonoids is considerably elevated, which we ascribe to the important role of this class of compounds in regulating flower development and fertilization. Indeed, in many plant species flavonoids are required for the proper development of stamens and germination of pollen tubes (Pollak et al., 1993; Muhlemann et al., 2018; Wang et al., 2020), a physiological function of flavonoids that has recently been discovered also in Arabidopsis (Zhang et al., 2021). Here, it has been shown that flavonoids protect toward the damaging effect of ROS, which fail to reach a harmful concentration due to scavenging activity of kaempferol derivatives. Of particular relevance is the observation that the content of flavonoids in Arabidopsis flowers is directly linked to plant fitness, as flavonoids’ deficient mutants when treated with kaempferol produce siliques with more seeds (Zhang et al., 2021).

Elevated levels of polyamines were detected only in very young florets, as their content progressively decreased with time and reached the lowest level in postanthesis (Figure 3). This general trend observed for all polyamine compounds is probably linked to their role in the process of pollen development as polyamines, by forming cross-links with different polymers, contribute to the stiffness of the pollen cell wall (Aloisi et al., 2016). After pollination, polyamines still contribute to pollen tube emergence but only as signaling molecules, for which the low levels we measured in our experiment are legitimate. In fact, high polyamine concentration in postanthesis would alter the morphology of the growing pollen tubes impairing reproduction (Rodriguez-Enriquez et al., 2013).

Indole and aliphatic glucosinolates showed dramatic differences across anthesis, with some compounds showing stable content and other displaying considerable variation as flower open (Figure 3). The physiological function of glucosinolates is related to plant defense, however, a pleiotropic effect of glucosinolate encoding genes on flowering time has also been proposed (Jensen et al., 2015; Kerwin et al., 2015). Sarsby et al. (2012) utilized mass spectrometry imaging to visualize aliphatic and indole glucosinolates in Arabidopsis flowers. In their studies, they measured variable concentrations of glucosinolates in different floral organs with peaks of intensity in sepals of young floral buds, in the floral calix specifically in the cells at the periphery of the phloem, and the siliques. Given their high content of sugars and amino acids floral tissues are highly palatable for which the role of glucosinolates as deterrent of herbivores has been proposed also in flowers.

### Transcripts associated with pathways of primary metabolisms and metabolites’ transport attend changes of primary metabolite content throughout anthesis

Elevated levels of transcripts of multiple genes associated with pathways of primary metabolism were detected across anthesis. Indeed, the KEGG categories of glycolysis, sucrose and starch metabolism, and TCA cycle were highly represented in each of the eight time points during which we sampled florets (Supplemental Figure S4). Comparative analyses between transcriptomic and metabolomic data, and the multiple positive correlations that we measured between metabolites and transcripts (Figure 7, A and B) suggest that the changes measured at the metabolite level are initiated at the transcriptional level. Besides, transcriptomic data also show that the high metabolic status perduring across anthesis is maintained by flowers through differential expression of set of genes which nevertheless belong to equivalent functional groups. For examples, genes in the categories of transmembrane transport of organic and inorganic solutes, such as ions, peptides, organic acids, and sugars are equally represented within the groups of transcripts showing either decreased or increased accretion across flower opening. However, the individual genes within each of these subgroups differ from one another (Figure 6, B and C). A plausible explanation for this happening is in the peculiar anatomy of flowers where the paucity of direct connections via the symplast resulted in the evolution of multiple tissue specific transporters with affinity for similar substrates (Borghi and Fernie, 2017). Therefore, we can speculate that at the time when individual floral organs develop and grow, their associated transporters and metabolic enzymes are transcriptionally turned on, and later, as tissues reach maturity and progressively age, turned off. Examples of these patterns can be seen when transcriptional data are utilized to follow the developmental progression of androecium, gynoecium, and finally the development of seeds and fruits (Pearce et al., 2015; Kivivirta et al., 2021).

The correlation network computed from all metabolites measured across anthesis identified GA and JA as relevant nodes connecting the majority of TCA intermediates (malate, citramalate, citrate, isocitrate, and glycerate), sugar phosphates (G6P and F6P) and sugar alcohols, amino acids (Glu, Gln, Asp, and Pro) and related polyamines, spermidine and putrescine (Figure 5). It is known that during the maturation phase of flower development, GA and auxin responsive factors promote the biosynthesis of JA and altogether regulate genes promoting elongation of petals and anthers, anther dehiscence, maturation of the gynoecium, and nectar secretion (Reeves et al., 2012; Cecchetti et al., 2013; Wiesen et al., 2016; Cardarelli and Costantino, 2018). The direct role of JA on the chemistry of flowers was initially discovered in Arabidopsis, where reduced emission of sesquiterpene volatiles was measured in multiple mutants along the pathway of JA synthesis and response (Reeves et al., 2012). In *Nattenuata*, where emission of scent and secretion of nectar are also regulated by JA, remarkable alterations of carbohydrate and energy metabolism were measured in the corolla of mutant lines with interrupted JA biosynthesis or perception (Stitz et al., 2014). Microarray experiments performed on corolla limbs of these lines showed overrepresentation of genes associated with glycolysis, metabolism of carbohydrates, and TCA cycle, resulting in altered accumulations of sugars and TCA intermediates. Interestingly, treatment with coronatine (a JA-Ile functional homolog) restored the chemical phenotype observed in wild-type corollas to a large extent. GA levels also have an impact on floral metabolism. Indeed, soon after pollination in the early stages of fruit set, GA rewires the pathways of central metabolism via transcriptional control of enzymes, plasma membrane and subcellular (organelle) transporters (Shinozaki et al., 2020). Moreover, strong correlations were measured among the levels of transcripts, proteins, and metabolites which may indicate a highly coordinated process taking place soon after pollination had occurred. Although these experiments were performed in tomato, comparison of gene expression atlases of plant organs across different species revealed that flower transcriptomes are all very similar to one another and therefore highly conserved (Julca et al., 2021). As it has been observed in *N.attenuata* and tomato, where the progression through the maturation phase of flower development and early fruit set is achieved via remobilization of carbohydrates and intermediates of the central pathways, similarly, in Arabidopsis, we detected tight correlations between the hub of genes associated with development and the genes associated with metabolism (Figure 6A). Whether metabolism is regulated in space and time via transcription, and vice-versa, we cannot ascertain from our dataset. However, evidence is slowly emerging suggesting that metabolism could also directly affect development (Miyazawa and Aulehla, 2018). Therefore, we cannot exclude that a reciprocal interdependency between metabolism and development may also take place in flowers.

Altogether, our study revealed that the process of flower opening is extensively sustained by temporal regulation of the central metabolic pathways. Indeed, transcripts and metabolites associated with glycolysis, carbohydrate metabolism, and TCA cycle are highly represented across flower anthesis, and while in preanthesis metabolism is rewired toward the synthesis of amino acids, those are later recycled to sustain embryo development. Studies of flower metabolism in Arabidopsis have so far been carried out on entire inflorescences; therefore, this study, which was conducted on individual florets, represents a substantial advancement in understanding flower metabolism. Nonetheless, the results of this study also suggest that specific clusters of metabolites and genes may support the temporal development of floral organs, as their physiological function changes across anthesis. Therefore, we acknowledge that substantial advancement will be gained from similar studies performed on individual floral organs, for which the data presented here will be of great support. Moreover, while it is commonly accepted that these metabolic changes are directed by the developmental progression of flowers across the maturation phase, it remains to be determined to what extent metabolism itself can affect development.

## Materials and methods

### Plant material and growth conditions

Seeds of Arabidopsis (*A.thaliana*) ecotype Col-0 (N6000) were stratified at 4°C in the dark for 5 days, sown in trays of 48 round pots (diameter 5 cm) with one plant per pot. Approximately 1,000 Arabidopsis plants were grown in a climate chamber (photoperiod: light/dark, 15/9 h; temperature 22°C/18°C, day/night; light intensity: 125 µMol^.^m^−2.^s^−1^) and bottom watered twice a week with 0.25× Hyponex solution alternated with deionized water until the plants bolted. Pools of ∼40 individual florets in eight consecutive stages of development (from S9 to S16) were harvested from the main bolting inflorescence and utilized for the analyses of metabolites and transcripts. All florets were harvested at around mid-day when mature flowers are fully open.

### Metabolite analysis

#### Analysis of primary and secondary metabolites

About 30–40 individual flowers (∼30 mg fresh weight [FW]) were harvested, promptly frozen in liquid nitrogen, and ground to a fine powder. Primary metabolites were extracted, annotated, and quantified following the procedure described by Lisec et al. (2006) and Alseekh et al. (2021). The protocols described by Tohge and Fernie (2010) and Perez de Souza et al. (2021) were followed to annotate secondary metabolites.

#### Analysis of apocarotenoids and hormones

Deuterated apocarotenoids (Buchem, Netherlands) and deuterated hormones (OlChemIm, Olomouc, Czech Republic) were used as internal standards 1 (IS-1) and IS-2 for the quantification of apocarotenoids and hormones, respectively. IS-1 mixture includes D_3_-β-apo-9-carotenone, D_3_-β-apo-13-carotenone, D_3_-β-apo-15-carotenal, D_3_-β-apo-14′-carotenal, D_3_-β-apo-12′-carotenal, D_3_-β-apo-10′-carotenal, D_3_-β-apo-8′-carotenal, and D_3_-3-OH-β-apo-13-carotenone. IS-2 mixture composed of D_6_-ABA, D_2_-JA, D_2_-GA3, D_2_-GA4, D_2_-GA9, D_2_-GA12, and D_2_-GA20. Approximately 10 mg FW Arabidopsis flowers were extracted with 1 mL of ethyl acetate with IS-1 in an ultrasound bath (Branson 3510 ultrasonic bath) for 15 min. After 8 min centrifugation at 2,163 *g* at 4°C, the supernatant was collected and the pellet was extracted with 1 mL of methanol with IS-2. Next, the two supernatants were combined and dried under vacuum. The residue was re-dissolved in 150 μL of methanol and filtered through a 0.22-μm filter before LC–MS analysis. Analysis of apocarotenoids and hormones was performed by using a HPLC (Agilent Technologies 1200, Germany) coupled to a Q-TRAP 5500 MS/MS (AB SCIEX, Framingham, MA, USA) with an electrospray source. Chromatographic separation of apocarotenoids was carried out on a ZORBAX Eclipse Plus C_18_ (150 × 2.1 mm, 3.5 μm) column with mobile phases of water (A) and acetonitrile (B) both containing 0.1% (v/v) formic acid. The gradient program was 0–20 min, 25%–100% B; 20–35 min, 100% B; 35–36 min, 100%–25% B; and 36–40 min, 25% B. The column was maintained at 35°C and the flow rate was 0.2 mL/min. The MS parameters for detection of apocarotenoids were as follows: positive ionization mode, temperature, 400°C; ion source gas 1, 80 psi; ion source gas 2, 70 psi; ion spray voltage, 5,500 V; curtain gas, 20 psi; and collision gas, medium. In addition, Chromatographic separation of hormones was carried out on a ZORBAX Eclipse Plus C_18_ (150 × 2.1 mm, 3.5 μm) column with mobile phases of water (A) and methanol (B) both containing 0.1% (v/v) formic acid. The gradient program was 0–18 min, 20%–100% B; 18–22 min, 100% B; 22–23 min, 100%–20% B; and 23–30 min, 20% B. The column was maintained at 40°C and the flow rate was 0.25 mL/min. The MS parameters for detection of hormones were as follows: negative ionization mode, temperature, 500°C; ion source gas 1, 45 psi; ion source gas 2, 30 psi; ion spray voltage, −4,500 V; curtain gas, 25 psi; and collision gas, medium. Annotation, Multiple Reaction Monitoring (MRM) transitions, and MS parameters of labeled and endogenous apocarotenoids and hormones are shown in Supplemental Table S2 (Mi et al., 2018). Data were acquired and analyzed using Analyst 1.6.2 software (Applied Biosystems).

#### VOCs trapping and analysis

Approximately 40 individual florets per stage of floral development were detached from the main inflorescence of Arabidopsis plants and enclosed in 50 mL clear glass vials sealed with aluminum/PTFE septum and kept in the same conditions of plant growth. VOCs were trapped for 2 h on a Tenax tube (Camsco, Texas, USA) connected with a syringe needle to the glass vial containing the flowers. Clean air filtered through a Tenax tube was pumped in the glass vial at a flow rate of 100 mL^.^min^−1^ with a manual vacuum pump (Pas-500 Personal Air sampler, Spectrex, Redwood City, California, USA). Volatiles were desorbed in a TD-100 thermal desorption instrument (Markes, UK) for 5 min at 240°C and concentrated on a cold trap at 0°C before the analysis. Volatiles were analyzed in a 7890B gas chromatography (GC) system set in split-less mode and equipped with 7200 Accurate-Mass quadrupole time-of-flight (Q-TOF) detector (Agilent Technologies, Santa Clara, California, USA). Separation of volatiles was performed on a DB5 capillary column with the following method: oven initial temperature 40°C for 2 min; ramp to 280°C at 10°C per minute; and postrun at 325°C for 2 min. Pure helium was used as a carrier gas at a rate of 1.5 mL per minute at the nominal pressure of 16 psi. GC-QToF operating conditions were set as described in Zhang et al. (2020). Peaks of known VOCs emitted by Arabidopsis flowers (Chen et al., 2003; Tholl et al., 2005; Zhang et al., 2020) were manually annotated and integrated upon comparison with library standards.

#### Metabolite visualization and plotting

Metabolite data were initially stored in Microsoft Excel and successively analyzed in R (version 3.4.3). The distribution of flower metabolite abundance in relation to the stage of development was initially explored with a principal component analysis performed in R with the “prcomp” function after centering and scaling, and plotted using the “plot” function. “tidyr” and “dplyr” (Wickham et al., 2019) were used to summarize *t* test statistics and “ggplot2” utilized to visualize the data with the function “ggboxplot” (Wickham, 2009). Correlations were computed using the R package Hmisc. Heatmaps were produced in R with the package “pheatmap” (Kolde and Kolde, 2015) after centering and scaling log2 average values of metabolite data. Cytoscape version 3.8.2 was used for network building.

### RNA isolation, analysis, and visualization

About 30–40 Arabidopsis florets in each of the eight stages of development (∼30 mg FW) were used as starting material for the isolation of the total RNA with Trizol Plus RNA purification kit and DNA digestion on column with PureLink DNase (Thermo Fisher Scientific, Waltham, Massachusetts, USA). Three samples of total RNA per stage of development were utilized for cDNA library construction and sequencing (Bioscience, WUR, Wageningen, The Netherlands). Transcriptome libraries were constructed using the TruSeq RNA sample Prep Kit (Illumina, San Diego, California, USA) and sequenced using the Illumina HiSeq2500 (Illumina). Trimmomatic (Bolger et al., 2014) was used to remove adaptor sequences, empty reads, short reads (<25 bp), reads with an N-ratio >10%, and low-quality sequences. Read counts were analyzed in R with the packages “EdgeR” and “limma” (Law et al., 2016, 2014; Bolger et al., 2014). “voom” normalization in limma was performed on those genes that fit a second degree quadratic polynomial (package “splines”; df = 3), following the assumption that changes occurring across development are smooth, gradual, and progressive. GO analysis was performed with the public server g:Profiler (Reimand et al., 2016). Venn diagrams were visualized with the R package “VennDiagram” (Chen and Boutros, 2011). K-mean cluster analysis was performed with the M:fuzz package (Kumar and Futschik, 2007). The list of genes with experimental and computational evidence of association to known metabolic pathways was downloaded from the Plant Metabolic Network database (Schlapfer et al., 2017) and the level of transcript abundance in the current dataset extracted and visualized on a heatmap as described in Borghi et al. (2019).

### Analysis of [U-^14^C]-Glc labeled samples

Radioactive isotope labeling was conducted for 5 h in a glass beaker sealed with Parafilm plastic and containing whole Arabidopsis inflorescences from which the siliques were removed. The inflorescences were standing upright in a 2 cm tall glass vial containing a solution of 100 mM K_2_SO_4_ and 100 mM Glc with specific activity of 7 MBq [U-^14^C]-Glc mol^−1^. To trap the labeled CO_2_ emitted from flowers a small plastic tube containing 0.5 mL of 1 M KOH was hung from the rim of the beaker and the solution retrieved with a syringe needle pierced through the seal of Parafilm plastic. A fluorescent lamp placed on top of the beakers was used as a source of light for all the duration of the experiment to replicate a greenhouse setting. The incorporation of radiolabeled carbon into the major classes of chemical compounds was determined after fractionation by ion-exchange chromatography coupled with enzymatic digestion in five biological replicates each constituted of three inflorescences. A detailed description of the reagents and method is provided by Obata et al. (2017). Metabolic fluxes were calculated following the assumptions described in Geigenberger et al. (1997, 2000).

### Analysis of [U-^13^C]-Glc labeled samples

About 2.5-cm tall inflorescences were cut from the main bolting stem of Arabidopsis plants and vertically placed in wells of a PCR plate (1 inflorescence per well) filled with a solution of 100 mM K_2_SO_4_ and 100 mM [U-^13^C]-Glc and kept in the greenhouse for the entire duration of the experiment. After 5 h of feeding, individual florets in the stages of preanthesis, anthesis, and postanthesis were harvested, snap-frozen in liquid nitrogen and stored at −80°C until further processing (*n* = 3; each sample being composed of multiple florets in one of the three stages of development). Primary metabolites were extracted and analyzed via GC–MS as previously described. Redistribution of ^13^C isotope was determined as described by Roessner-Tunali et al. (2004) and Lima et al. (2018).

## Supplemental data

The following materials are available in the online version of this article.

**Supplemental Figure S1.** Heatmap of metabolite–metabolite correlations across flower anthesis.

**Supplemental Figure S2.** Multidimensional plot of log normalized counts.

**Supplemental Figure S3.** GO term enrichment of DEGs in the developmental transitions of *Athaliana* flowers across anthesis.

**Supplemental Figure S4.** KEGG term enrichment of DEGs in the developmental transitions of *A.thaliana* flowers across anthesis.

**Supplemental Figure S5.** Venn diagrams and top category of GO/ GO/KEGG term enrichment of DEGs from *A.thaliana* florets across anthesis.

**Supplemental Figure S6.** Heatmaps of log_2_ normalized transcripts of genes with known experimental and computational association with pathways of central and secondary metabolism across flower anthesis.

**Supplemental Figure S7.** Gene-metabolite correlation networks.

**Supplemental Table S1.** Total amount of metabolites in individual florets following [U-^13^C]-Glc feeding.

**Supplemental Table S2.** MRM transitions and MS parameters of labeled and endogenous apocarotenoids and hormones.

**Supplemental Data Set S1.** Primary and secondary metabolites and hormones measured in *A.thaliana* florets in different stages of development across anthesis.

**Supplemental Data Set S2.** Pearson pairwise metabolite to metabolite correlations.

**Supplemental Data Set S3.** Log_2_ normalized values of transcripts from DEGs in *A.thaliana* flowerets in eight consecutive stages of development across anthesis.

**Supplemental Data Set S4.** DEG and GO Term enrichment analysis.

**Supplemental Data Set S5.** GO term enrichment of genes in the functional clusters “development,” “metabolism,” and “signaling” shown in Figure 6.

**Supplemental File S1.** Network of correlative associations across transcripts (Figure 6A; Cytoscape file).

**Supplemental File S2.** Correlation network between metabolites and transcripts in preanthesis (Figure 7A; Cytoscape file).

**Supplemental File S3.** Correlation network between metabolites and transcripts in postanthesis (Figure 7B; Cytoscape file).

## Supplementary Material

kiac253_Supplementary_Data

## Notes

M.B. conceived and designed the study with inputs from H.J.B. and A.R.F. M.B. performed the experiments. M.B., T.T., and J.M. analyzed the metabolites. M.B. and L.P.d.S. analyzed the redistribution of ^13^C. M.B. and M.M. performed and analyzed the redistribution of ^14^C. M.B. analyzed transcripts with inputs from L.P.d.S., G.M., and S.P. M.B. wrote the manuscript with inputs from A.R.F. S.A.-B., H.J.B., and A.R.F. provided reagents and supervision.

The authors responsible for distribution of materials integral to the findings presented in this article in accordance with the policy described in the Instructions for Authors (https://academic.oup.com/plphys/pages/general-instructions) are: Harro J. Bouwmeester (H.J.Bouwmeester@uva.nl) and Alisdair R. Fernie (Fernie@mpimp-golm.mpg.de).
