# Supplementary material for: High-energy-level metabolism and transport occur at the transition from closed to open flowers
Source: Plant Physiol. 2022 May 28;190(1):319–39. doi: 10.1093/plphys/kiac253 (PMC9434183; doi:10.1093/plphys/kiac253)
Supplement: kiac253_Supplementary_Data [file kiac253_supplementary_data.zip › Supplemental_FiguresXTables.pdf]

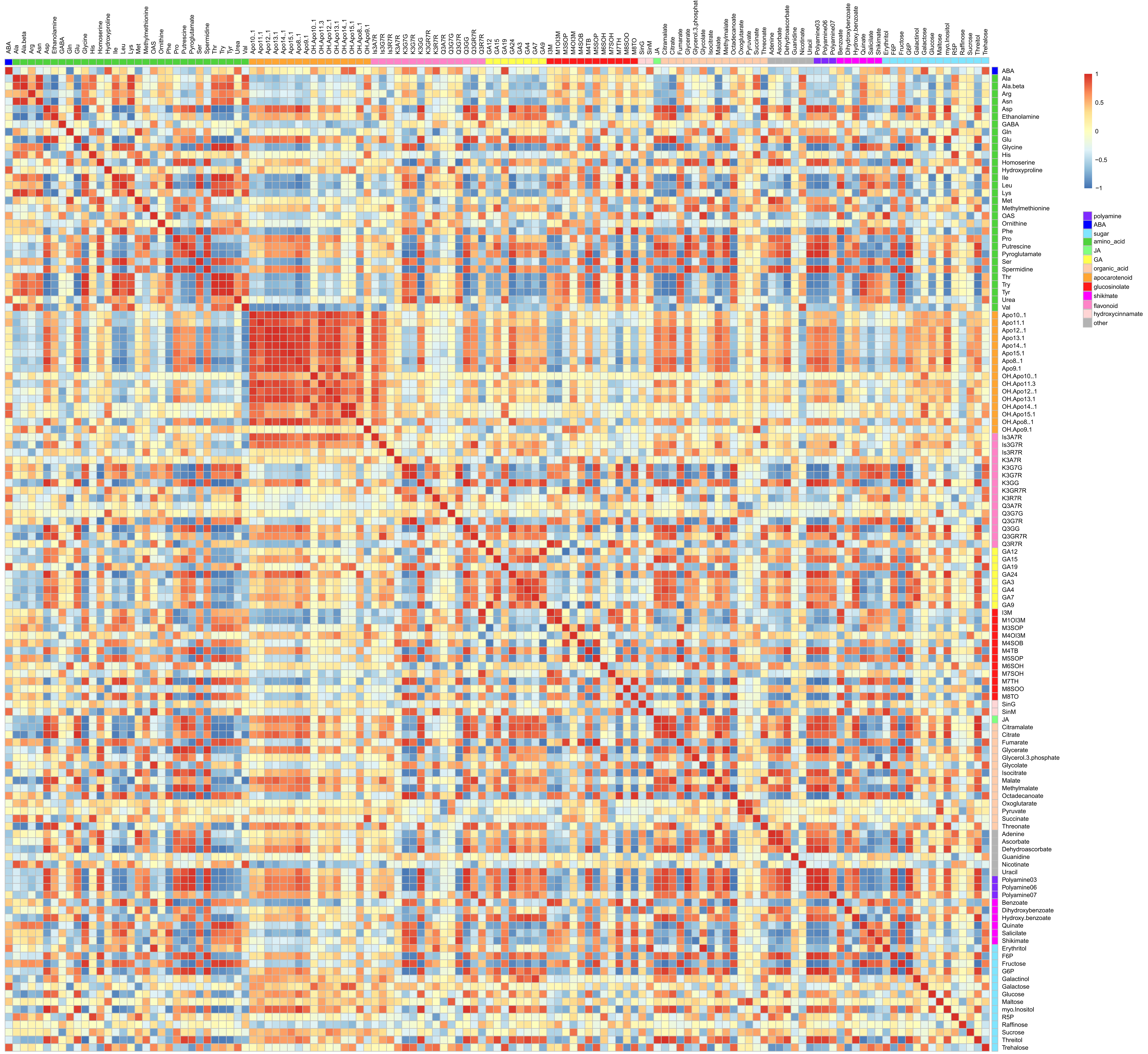

Supplemental Figure S1. Heatmap of metabolite-metabolite correlations across flower anthesis. Metabolites are color-coded accordingly to their association with the following biosynthetic pathways: deep purple, polyamine; blue, abscisic acid (ABA); light blue, sugar and starch; dark green, amino acids; light green, jasmonic acid (JA); yellow, gibberellins (GA); tan, organic acids; orange, apocarotenoids; red, glucosinolates; magenta, shikimate; lilac, flavonoid; peach rosé, hydroxycinnamates; grey, others.

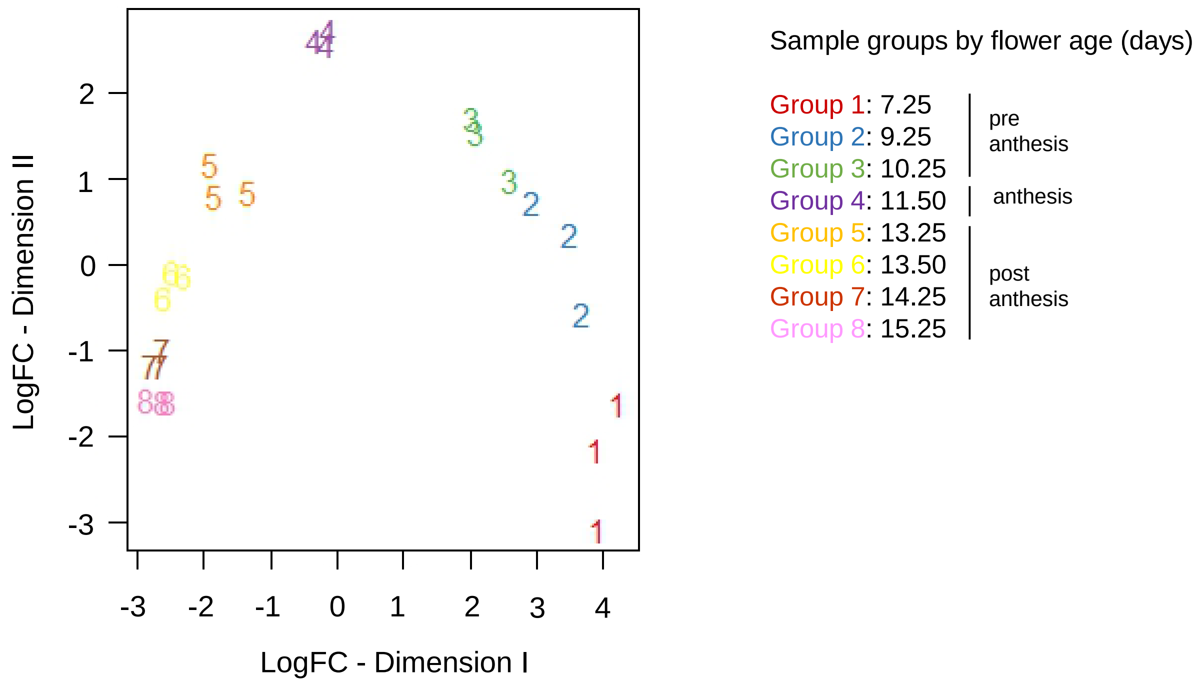

Supplemental Figure S2. Multidimensional plot of log of normalized counts. Distances on the plot correspond to the leading fold change (FC), which is the average log2 FC for the most divergent genes between each pair of samples. Sample groups correspond to florets in different stages of development (flower age in days).

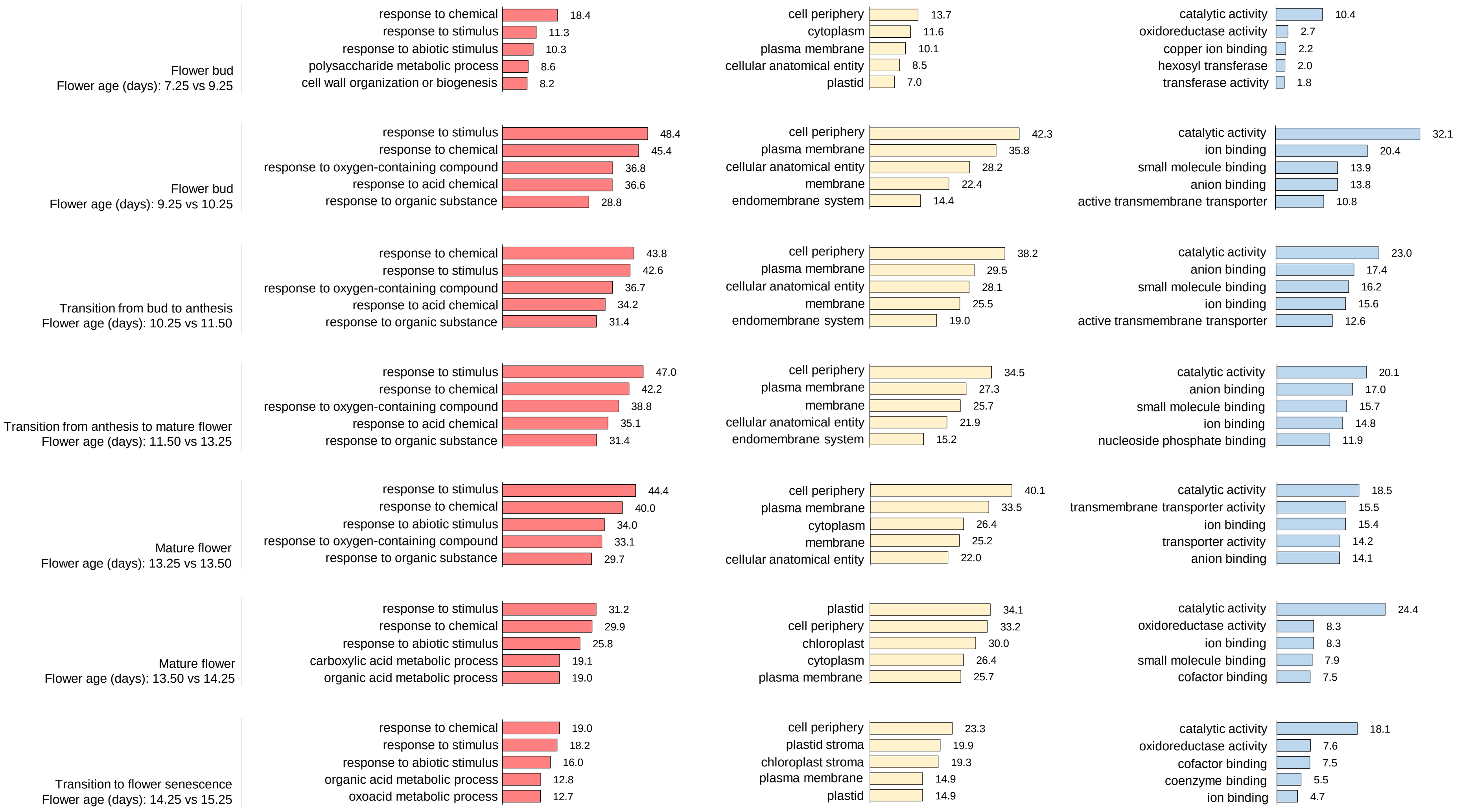

Supplemental Figure S3 Gene Ontology (GO) term enrichment of differentially expressed genes in the developmental transitions of *Arabidopsis thaliana* flowers across anthesis. The figure shows negative Log_{10} of the adjusted P value of the first five enriched terms in the GO categories biological process (red bars), cellular component (yellow), and molecular function (blue).

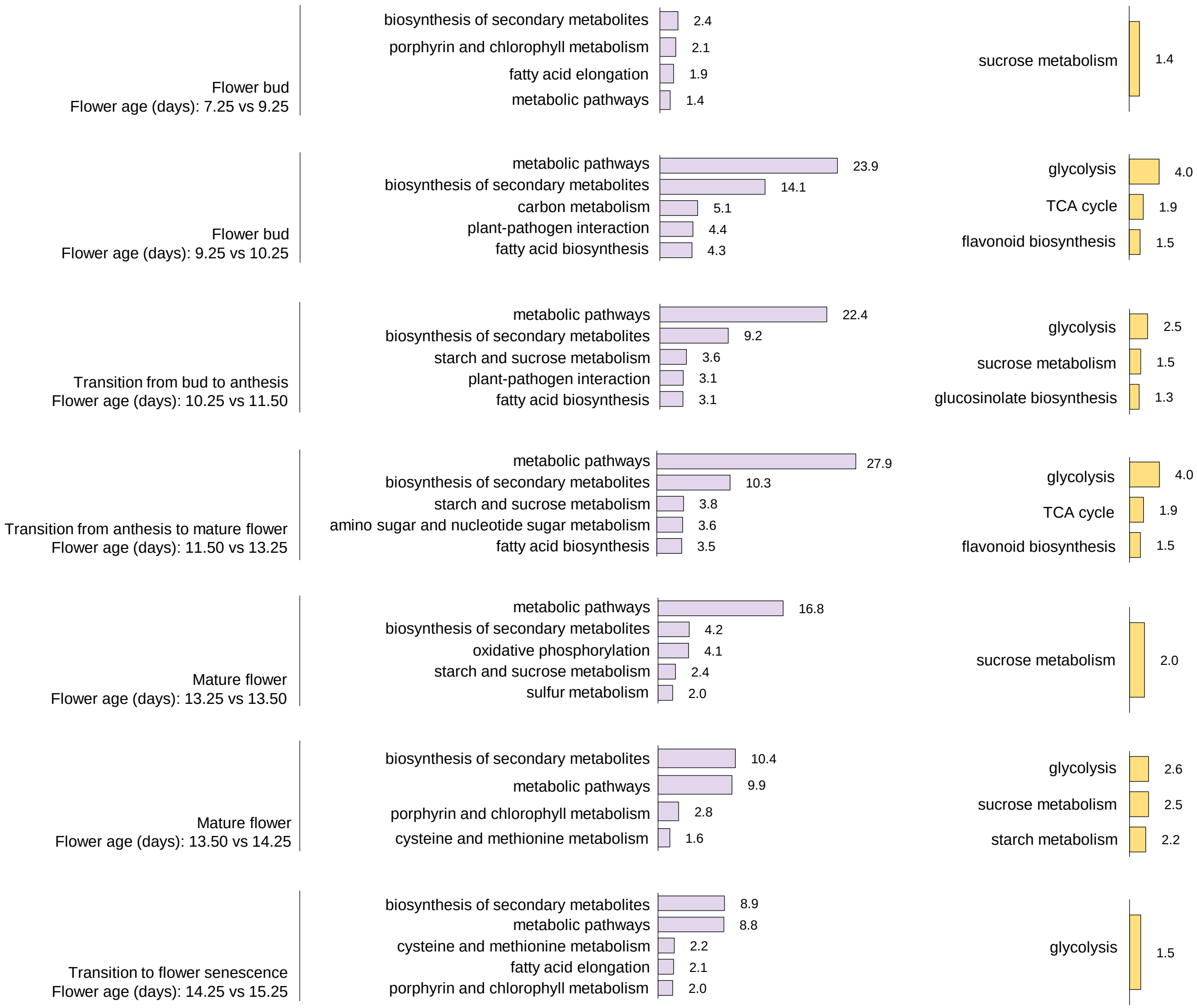

Supplemental Figure S4. Kyoto Encyclopedia of Genes and Genomes (KEGG) term enrichment of differentially expressed genes in the developmental transitions of *Arabidopsis thaliana* flowers across anthesis. The figure shows negative $\log_{10}$ of the adjusted P value of the main KEGG terms (lilac) and most represented metabolic pathways in the top KEGG category (yellow bars).

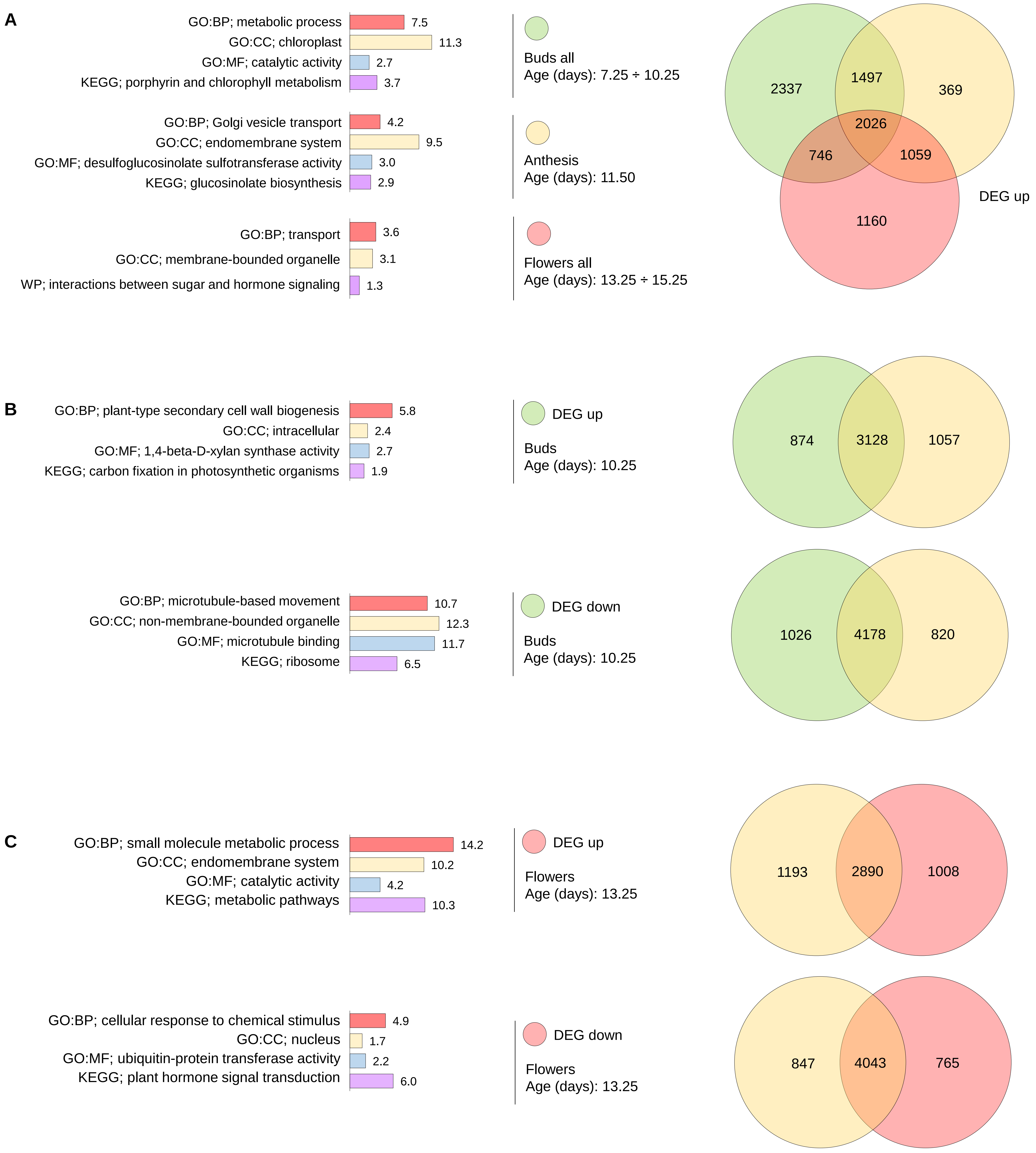

Supplemental Figure S5. Venn diagrams and top categories of Gene Ontology/Kyoto Encyclopedia of Genes and Genomes (GO/KEGG) term enrichment of differentially expressed genes (DEGs) from *Arabidopsis thaliana* florets across anthesis. A, DEGs from flowers in pre-anthesis (buds from age 7.25 to 10.25 days; green color), anthesis (yellow), and post-anthesis (mature flowers from age 13.25 to 15.25 days; pink). DEGs from (B) transition from buds (10.25 days of age; green) to anthesis (11.50 days of age; yellow) and (C) from anthesis (11.50 days of age; yellow) to mature flowers (13.35 days of age; red). Abbreviations: BP, biological process; CC, cellular component; MF, molecular function.

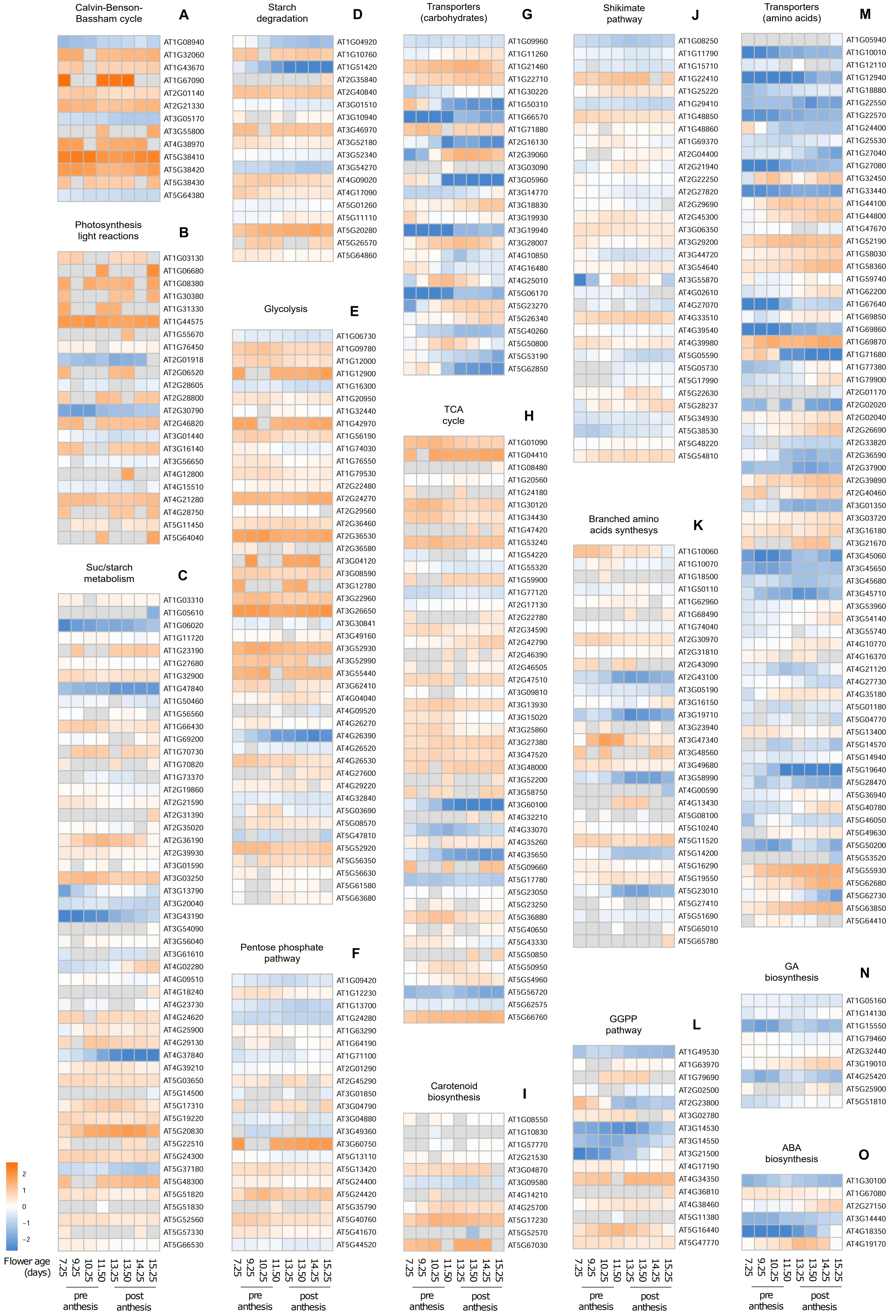

Supplemental Figure S6. Heat-maps of $\log_2$ normalized transcripts of genes with known experimental and computational association with pathways of central and specialized metabolism across flower anthesis. Only genes that are significant (p.adj < 0.05) in at least one of the possible contrasts and that fit a quadratic regression are shown in the heat-maps. The following group-pathway associations are shown: A, Calvin-Benson-Bassham cycle; B, photosynthesis light reactions; C, sucrose and starch metabolism; D, starch degradation and sucrose biosynthesis; E, glycolysis; F, pentose phosphate pathway; G, carbohydrate transporters; H, TCA cycle including pyruvate decarboxylation to acetyl CoA synthesis; I, carotenoid biosynthesis; J, shikimate pathway to aromatic amino acid synthesis; K, branched chain amino acid synthesis; L, GGPP biosynthesis (via MEP pathway); M, transporters of amino acids, peptides, and nitrate; N, GA biosynthesis via entkaurene synthase; O, biosynthesis of ABA.

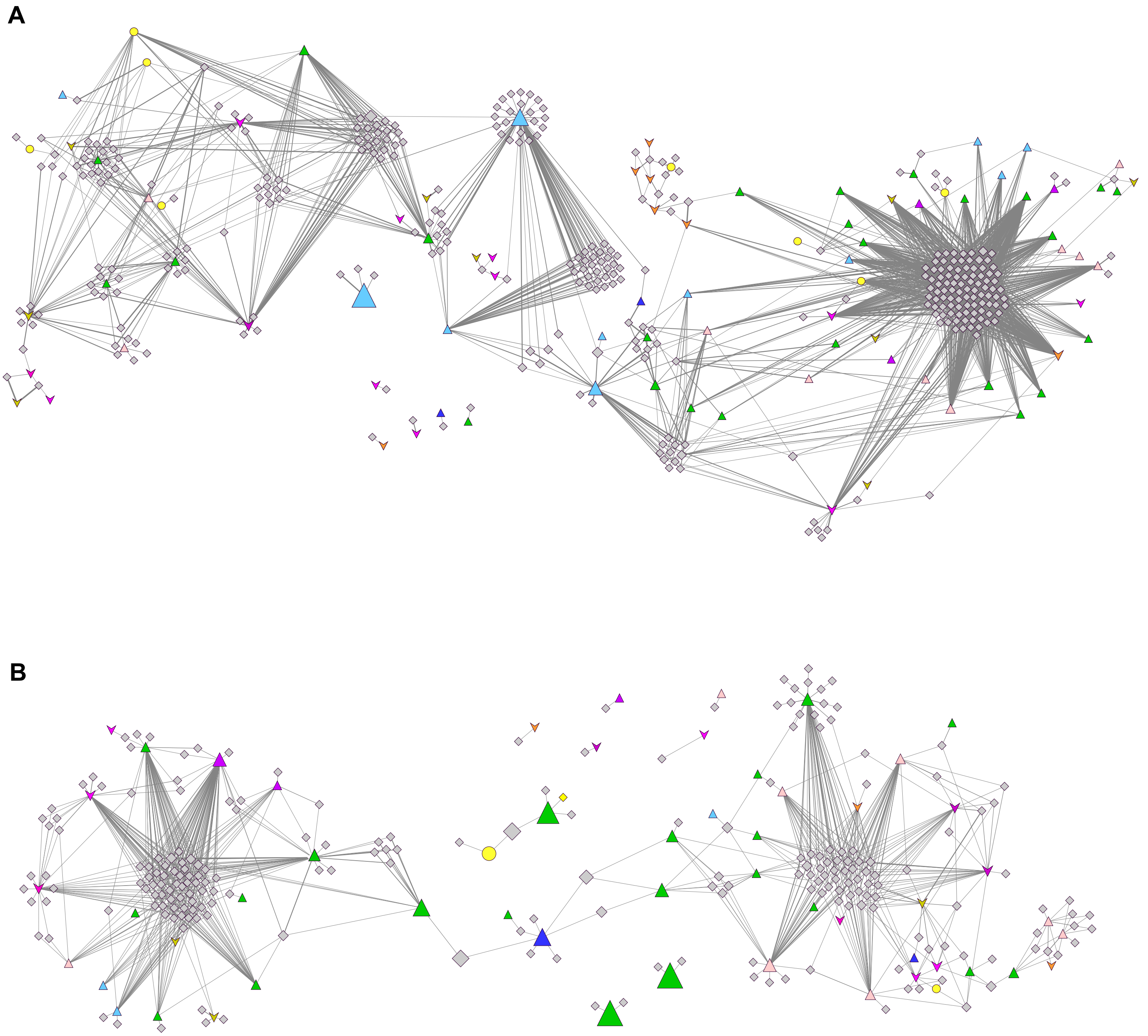

Supplemental Figure S7. Gene-metabolite correlation networks. A, visualization of the Pearson correlations between transcripts and metabolites in Arabidopsis florets in developmental stages from S9 to S13 (pre-anthesis) and B, developmental stages from S13 to S16 (post-anthesis). Genes are represented with grey diamond symbols, primary metabolites with triangles, secondary metabolites with inverted triangles, and hormones with circles. Amino acids are colored in green, carbohydrates in light blue, organic acids in pink, apocarotenoids in orange, metabolites of the shikimate pathway in purple, flavanols, hydroxycinnamates, and polyamines with different shades of purple-violet, glucosinolates in cress-green. Other metabolites are represented in deep blue. The correlation matrix was computed using the R package Hmisc and represented with an organic layout in Cytoscape 3.8.2. Spring length and mass was manually adjusted to avoid node overlapping. The size of nodes is proportional to the network parameter of betweeness centrality, with larger nodes representing highly connected metabolites and genes in the network. Edge thickness is proportional to the correlation value with thicker edges representing stronger correlations. Only positive correlation above the value of 0.80 are represented in the figure.

Supplemental Table S1. Total amount of metabolites in individual florets following [U-^{13}C]-Glc feeding of whole *Arabidopsis thaliana* inflorescences. Inflorescences were cut from the main bolting stem of flowering plants and incubated in a solution of 100 mM K_2SO_4 and 100 mM [U-^{13}C]-Glc for 5 hours. At the end of the incubation period, florets in the stage of pre-anthesis, anthesis, and post-anthesis were separately harvested, metabolites extracted, and the total amount of metabolites quantified via GC-MS. Values represent the average (ng mg^{-1} FW) of 3 samples ± SE. Different letters indicate values significantly different at *P < 0.05*, while metabolites in bold are significantly different at *P < 0.01* in at least one of three possible pairwise comparisons (T-test).

		Stage of development											
Metabolite	Group	pre-anthesis				anthesis				post-anthesis			
		avg		se		avg		se		avg		se	
Ala	Amino acid	457.54	±	64.80	a	872.92	±	100.12	b	901.43	±	73.86	b
Asn	Amino acid	2304.53	±	197.29	a	2844.42	±	521.83	a	1556.53	±	179.31	b
Asp	Amino acid	1045.94	±	101.87	a	834.12	±	96.66	a	429.74	±	20.14	b
GABA	Amino acid	295.43	±	35.00	a	394.73	±	2.65	b	269.68	±	19.02	a
Glu	Amino acid	7317.69	±	630.43	a	6972.18	±	817.17	a	3337.05	±	303.45	b
Glycine	Amino acid	394.85	±	29.54	a	632.83	±	59.57	b	1180.89	±	116.66	c
Ile	Amino acid	177.28	±	19.26	a	290.49	±	18.50	b	212.50	±	18.25	a
Leu	Amino acid	207.54	±	25.37	a	317.92	±	20.47	b	236.40	±	22.98	a
Ornithine	Amino acid	95.53	±	6.14	a	119.78	±	7.85	b	91.39	±	2.10	a
Pro	Amino acid	2048.94	±	259.22	a	3151.97	±	260.70	b	1598.75	±	102.29	c
Ser	Amino acid	715.30	±	88.65	a	942.78	±	138.66	b	1025.74	±	98.16	b
Thr	Amino acid	517.34	±	68.16	a	1072.57	±	122.41	b	893.67	±	122.76	b
Val	Amino acid	2685.26	±	366.92	a	3861.40	±	338.19	b	2445.85	±	294.64	a
**Fru**	**Carbohydrate**	**831.01**	**±**	**125.53**	**a**	**8685.44**	**±**	**593.95**	**b**	**13851.54**	**±**	**1309.78**	**c**
**Glc**	**Carbohydrate**	**196941.06**	**±**	**17388.47**	**a**	**614589.46**	**±**	**9388.14**	**b**	**427751.69**	**±**	**46095.80**	**c**
**Myoinositol**	**Carbohydrate**	**2741.82**	**±**	**327.42**	**a**	**3979.27**	**±**	**19.52**	**b**	**2459.10**	**±**	**147.80**	**a**
Suc	Carbohydrates	14714.29	±	1966.66	a	26383.15	±	1147.00	b	15325.96	±	836.97	a
Trehalose	Carbohydrates	362.28	±	30.56	a	489.97	±	2.48	b	395.84	±	14.67	a
**Citrate**	**Organic acid**	**4518.91**	**±**	**591.92**	**a**	**2177.92**	**±**	**29.45**	**b**	**1506.34**	**±**	**45.19**	**c**
**Fumarate**	**Organic acid**	**662.55**	**±**	**57.13**	**a**	**3014.39**	**±**	**357.79**	**b**	**2647.13**	**±**	**140.33**	**b**
Glycerate	Organic acid	69.61	±	10.80	a	140.58	±	7.71	b	87.80	±	20.51	a
Malate	Organic acid	3412.38	±	264.60	a	7327.13	±	362.62	b	3973.47	±	291.85	a
**Succinate**	**Organic acid**	**550.11**	**±**	**44.89**	**a**	**2246.35**	**±**	**186.83**	**b**	**996.03**	**±**	**61.49**	**c**

Supplemental Table S2. Multiple Reaction Monitoring (MRM) transitions and MS parameters of labeled and endogenous apocarotenoids and hormones.

Compounds ID	Abbreviation	Time (min)	Q1/Q3 (*m/z*)	DP (V)	CXP (V)	EP (V)	CE (V)	IS (ng per sample)
β-apo-9-carotenone	Apo-9	22.1	193.2 / 109.1	60	11	10	25	D_3-Apo-9 (2.4)
β-apo-11-carotenal	Apo-11	24.6	219.2 / 145.1	60	11	10	25	D_3-Apo-13 (2.3)
β-apo-13-carotenone	Apo-13	26.9	259.2 / 175.1	60	11	10	25	D_3-Apo-13 (2.3)
β-apo-15-carotenal	Apo-15	28.4	285.2 / 175.1	60	11	10	25	D_3-Apo-15 (2.9)
β-apo-14'-carotenal	Apo-14'	29.5	311.2 / 159.1	60	11	10	25	D_3-Apo-14' (2.2)
β-apo-12'-carotenal	Apo-12'	31.1	351.3 / 119.1	60	11	10	25	D_3-Apo-12' (2.4)
β-apo-10'-carotenal	Apo-10'	31.3	377.3 / 119.1	60	11	10	25	D_3-Apo-10' (3.8)
β-apo-8'-carotenal	Apo-8'	32.3	417.3 / 119.1	60	11	10	25	D_3-Apo-8' (4.2)
3-OH-β-apo-9-carotenone	OH-Apo-9	12.5	209.2 / 173.1	60	11	10	25	D_3-OH-Apo-13 (1.7)
3-OH-β-apo-11-carotenal	OH-Apo-11	15.3	235.2 / 173.1	60	11	10	25	D_3-OH-Apo-13 (1.7)
3-OH-β-apo-13-carotenone	OH-Apo-13	17.9	275.2 / 173.1	60	11	10	25	D_3-OH-Apo-13 (1.7)
3-OH-β-apo-15-carotenal	OH-Apo-15	20.1	301.2 / 173.1	60	11	10	25	D_3-OH-Apo-13 (1.7)
3-OH-β-apo-14'-carotenal	OH-Apo-14'	21.9	327.2 / 173.1	60	11	10	25	D_3-OH-Apo-13 (1.7)
3-OH-β-apo-12'-carotenal	OH-Apo-12'	24.8	367.3 / 173.1	60	11	10	25	D_3-OH-Apo-13 (1.7)
3-OH-β-apo-10'-carotenal	OH-Apo-10'	25.5	393.3 / 173.1	60	11	10	25	D_3-OH-Apo-13 (1.7)
3-OH-β-apo-8'-carotenal	OH-Apo-8'	27.5	433.3 / 173.1	60	11	10	25	D_3-OH-Apo-13 (1.7)
D_3-β-apo-9-carotenone	D3-Apo-9	22.1	196.2 / 178.1	60	11	10	25	_
D_3-β-apo-13-carotenone	D3-Apo-13	26.9	262.2 / 178.1	60	11	10	25	_
D_3-β-apo-15-carotenal	D3-Apo-15	28.4	288.2 / 178.1	60	11	10	25	_
D_3-β-apo-14'-carotenal	D3-Apo-14'	29.5	314.2 / 162.1	60	11	10	25	_
D_3-β-apo-12'-carotenal	D3-Apo-12'	31.1	354.3 / 119.1	60	11	10	25	_
D_3-β-apo-10'-carotenal	D3-Apo-10'	31.3	380.3 / 119.1	60	11	10	25	_
D_3-β-apo-8'-carotenal	D3-Apo-8'	32.3	420.3 / 119.1	60	11	10	25	_
D_3-3-OH-β-apo-13-carotenone	D3-OH-Apo-13	17.9	278.2 / 173.1	60	11	10	25	_
Gibberellin A3	GA3	8.7	345.1 / 239.0	-70	-10	-10	-30	D_2-GA3 (1.0)
Gibberellin A4	GA4	15.2	331.0 / 213.0	-70	-10	-10	-30	D_2-GA4 (1.0)
Gibberellin A7	GA7	14.8	329.1 / 223.0	-70	-10	-10	-30	D_2-GA4 (1.0)
Gibberellin A9	GA9	16.4	315.2 / 271.2	-70	-10	-10	-30	D_2-GA9 (1.0)
Gibberellin A12	GA12	18.4	331.2 / 313.0	-70	-10	-10	-30	D_2-GA12 (1.0)
Gibberellin A15	GA15	13.8	329.2 / 257.0	-70	-10	-10	-30	D_2-GA9 (1.0)
Gibberellin A19	GA19	13.8	361.2 / 273.5	-70	-10	-10	-30	D_2-GA20 (1.0)
Gibberellin A24	GA24	16.2	345.2 / 257.0	-70	-10	-10	-30	D_2-GA20 (1.0)
Abscisic acid	ABA	11.8	263.0 / 153.0	-25	-5	-10	-20	D_6-ABA (1.0)
Jasmonic acid	JA	13.3	209.0 / 59.0	-25	-5	-10	-25	D_2-JA (1.9)
D_2-Gibberellin A3	D_2-GA3	8.7	347.1 / 241.0	-70	-10	-10	-30	_
D_2-Gibberellin A4	D_2-GA4	15.2	333.0 / 259.0	-70	-10	-10	-30	_
D_2-Gibberellin A9	D_2-GA9	16.4	317.0 / 273.0	-70	-10	-10	-30	_
D_2-Gibberellin A12	D_2-GA12	18.4	333.2 / 315.0	-70	-10	-10	-30	_
D_2-Gibberellin A20	D_2-GA20	12.7	333.0 / 289.3	-70	-10	-10	-30	_
D_6-Abscisic acid	D_6-ABA	11.8	269.1 / 159.0	-25	-5	-10	-20	_
D_2-Jasmonic acid	D_2-JA	13.3	211.0 / 61.0	-25	-5	-10	-25	_
